# STForte: tissue context-specific encoding and consistency-aware spatial imputation for spatially resolved transcriptomics

**DOI:** 10.1093/bib/bbaf174

**Published:** 2025-04-21

**Authors:** Yuxuan Pang, Chunxuan Wang, Yao-zhong Zhang, Zhuo Wang, Seiya Imoto, Tzong-Yi Lee

**Affiliations:** Division of Health Medical Intelligence, Human Genome Center, The Institute of Medical Science, The University of Tokyo, 4-6-1, Shirokanedai, Minato-ku, Tokyo, 108-8639, Japan; School of Data Science, The Chinese University of Hong Kong, Shenzhen (CUHK-Shenzhen), 2001 Longxiang Road, Longgang, Shenzhen, 518172, China; Division of Health Medical Intelligence, Human Genome Center, The Institute of Medical Science, The University of Tokyo, 4-6-1, Shirokanedai, Minato-ku, Tokyo, 108-8639, Japan; Warshel Institute for Computational Biology, School of Medicine, The Chinese University of Hong Kong, Shenzhen (CUHK-Shenzhen), 2001 Longxiang Road, Longgang, Shenzhen, 518172, China; School of Medicine, The Chinese University of Hong Kong, Shenzhen (CUHK-Shenzhen), 2001 Longxiang Road, Longgang, Shenzhen, 518172, China; Division of Health Medical Intelligence, Human Genome Center, The Institute of Medical Science, The University of Tokyo, 4-6-1, Shirokanedai, Minato-ku, Tokyo, 108-8639, Japan; Institute of Bioinformatics and Systems Biology, National Yang Ming Chiao Tung University, No. 75 Bo-Ai Street, Hsinchu 300, Taiwan

**Keywords:** spatial transcriptomics, self-supervised learning, deep learning, imputation, graph autoencoder

## Abstract

Encoding spatially resolved transcriptomics (SRT) data serves to identify the biological semantics of RNA expression within the tissue while preserving spatial characteristics. Depending on the analytical scenario, one may focus on different contextual structures of tissues. For instance, anatomical regions reveal consistent patterns by focusing on spatial homogeneity, while elucidating complex tumor micro-environments requires more expression heterogeneity. However, current spatial encoding methods lack consideration of the tissue context. Meanwhile, most developed SRT technologies are still limited in providing exact patterns of intact tissues due to limitations such as low resolution or missed measurements. Here, we propose STForte, a novel pairwise graph autoencoder-based approach with cross-reconstruction and adversarial distribution matching, to model the spatial homogeneity and expression heterogeneity of SRT data. STForte extracts interpretable latent encodings, enabling downstream analysis by accurately portraying various tissue contexts. Moreover, STForte allows spatial imputation using only spatial consistency to restore the biological patterns of unobserved locations or low-quality cells, thereby providing fine-grained views to enhance the SRT analysis. Extensive evaluations of datasets under different scenarios and SRT platforms demonstrate that STForte is a scalable and versatile tool for providing enhanced insights into spatial data analysis.

## Introduction

To comprehend the functioning of life, it is crucial to observe the behavior of cells or groups of cells in multicellular organisms [1]. One approach to achieving this is through the use of spatially resolved transcriptomics (SRT) [2, 3]. It provides a detailed view for specifying diversity among different spatial regions, enabling the study of critical biological processes.

Recent advances in SRT have enabled researchers to test hypotheses and uncover insights through observation and exploratory data analysis (EDA) [4]. To this end, various computational methods have been proposed to facilitate the progress of EDA using SRT data. Specifically, latent encoding extraction and spatial region identification, which transform complex gene expression data into interpretable low-dimensional representations and meaningful spatial domains, are essential processes in SRT analysis that provide condensed information for subsequent downstream analyses. These methods can be based on statistical frameworks [5–7] or follow graph neural network architectures [8–12]. Most existing methods consider introducing spatial correlations between neighboring spots to induce spatial homogeneity [13]. Ascertaining spatial homogeneity in SRT analysis benefits related tasks in dissecting consecutive spatial domains, such as identifying anatomical regions. However, excessive consideration of spatial correlation may cause overemphasis on homogeneous and smooth information, resulting in the absence of heterogeneous patterns, such as tumor micro-environments (TMEs) [14–16].

SRT technologies can generally be categorized into two types [17]: imaging-based and next-generation sequencing (NGS)-based technologies. However, these methods are still restricted by their relatively low sensitivity and resolution. Specifically, despite recently flourished NGS-based approaches with increasingly higher spatial resolution in transcript measurements [18–21], some of the trending approaches [22, 23] have limited resolution and spatial coverage in capturing the transcripts of intact tissue. Resolution enhancement can be performed by combining histological images [24, 25] or by considering sub-spot resolutions using probabilistic approaches [6, 7]. Besides, another approach considers leveraging methods from computer vision, utilizing deep neural networks to directly predict expression values for predefined unobserved locations [26]. Nevertheless, the existing methods either depend on histological images and large computational resources or are deficient in flexibility and the consideration of structural contexts for spatial imputation.

Here, we propose STForte, a graph-learning-based autoencoding approach, to address the aforementioned challenges in SRT data analysis. STForte is crafted using a modified pairwise graph autoencoder (GAE), a neural network-based architecture that simultaneously handles the gene expression profiles and spatial topology of SRT data by manipulating their correspondence within the latent space. STForte highlights the following aspects. Firstly, modeling the correspondence enables the framework to adaptively capture the impartial patterns between the expression attributes and spatial correlation, which facilitates the capability in characterizing both the homogeneity and heterogeneity in SRT data. Secondly, the spatial topology is encoded to unify the spatial correlation, which can be used to impute the biological patterns of locations with unmeasured and deficient transcripts, which allows for obtaining more consecutive content and achieving fine-grained resolution. STForte is scalable to accommodate various analytical scenarios for SRT data. Experiments were conducted, demonstrating that STForte is applicable to various SRT data obtained from different platforms, and can be adapted for single-slice and multi-slice analysis.

## Results

### Overview of STForte method

**Figure 1:**
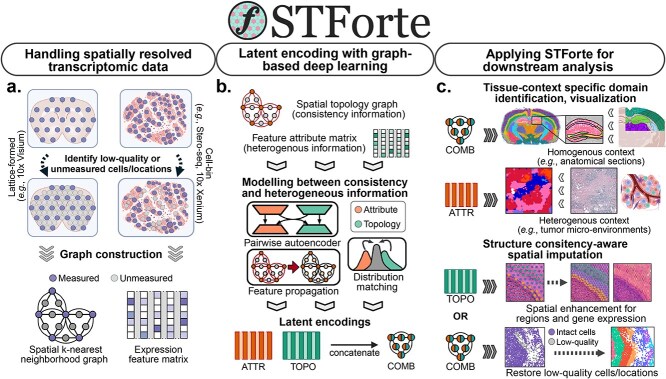
Schematic overview of our proposed approach. **a**, STForte processes SRT data to extract spatial KNN graph and expression feature matrix, identifying unmeasured or low-quality locations through automatic padding or user-defined procedures. **b**, The spatial graph contains consistency information while the feature attribute matrix captures expression heterogeneity, which are modeled and integrated using a pairwise autoencoder to learn latent encodings of feature attributes (ATTR) and spatial topology (TOPO), with adversarial distribution matching and feature propagation facilitating the modeling of association between these information types. **c**, The resulting encodings (ATTR, TOPO, and their concatenation COMB) can serve various analytical purposes. The illustration was created with BioRender.com.

A schematic overview of STForte is illustrated in Fig. 1, with technical details described in the Methods section and Supplementary Notes. Briefly, SRT data are processed to extract their topological relations and transcription profiles. For lattice-formed NGS-based SRT data protocols, such as 10x Visium, STForte can automatically generate padding spots from the original collection of spots based on their coordinates and a predefined distance. Users can also manually mask or insert spots for mismeasured or unmeasured locations to infer their biological patterns (Fig. 1a). A topological graph is subsequently established using spatial k-nearest neighbors (KNN) or their straightforward closeness based on a predefined distance. For the observed expression feature attributes, STForte can directly accept the count matrix or use the results obtained from the Principal Component Analysis (PCA).

STForte adopts a pairwise GAE-based framework (Fig. 1b and Supplementary Fig. S1) based on cross-reconstruction and adversarial distribution matching to jointly learn the latent space for both the expression attributes and spatial topology information of SRT data. The framework also enables STForte to infer the biological patterns of locations with inaccessible or mismeasured transcripts. In addition, a feature propagation technique is adopted to facilitate the inference of inaccessible locations for expression attributes. Subsequently, the attributes (ATTR), topology (TOPO) encodings, and their combined encodings by concatenation (COMB) can serve diverse downstream analyses, such as visualization, spatial region identification through clustering algorithms, and spatial imputation that infers biological patterns based on spatial consistency (Fig. 1c). Spatial imputation can be used to propagate spatial region annotations or predict expressions of unseen locations, which enables analysis and discovery under better resolution or in extra biological contexts.

### STForte facilitates analysis with refined resolution and consistent patterns on 10x Visium MOB data

**Figure 2:**
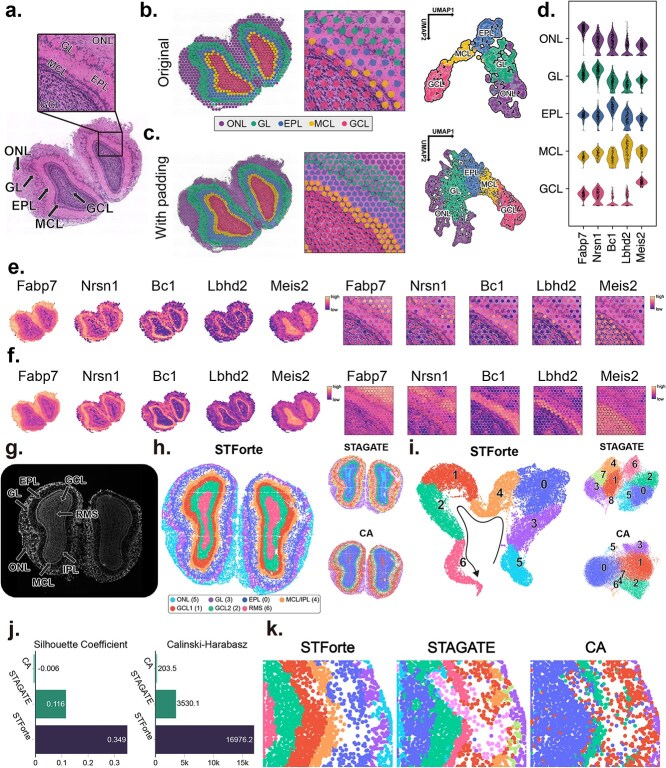
**a**, H&E-stained image and annotation of the 10x Visium MOB data including a zoomed-in region. **b**, Spatial regions and UMAP visualization obtained from STForte (COMB encoding). **c**, Spatial regions and UMAP visualization under padding views obtained from STForte (TOPO encoding). **d**, Violin plots of gene expression levels of the selected five layer-specific marker genes. **e**, Visualization of the spatial expression levels of five layer-specific genes on the entire dataset (left) and the zoomed-in region (right). **f**, Spatial expression levels after STForte’s padding on the entire dataset (left) and the zoomed-in region (right). **g**, DAPI-stained image with manual annotation of the Stereo-seq MOB data. **h**, Spatial regions identified by different dimensional reduction methods, including STForte, STAGATE, and CA. **i**, UMAP visualizations obtained by different methods. **j**, Comparison of clustering metrics for different methods. **k**, Zoomed-in region of interest showing the results of low-quality spots processed by different methods.

We first applied STForte to a mouse olfactory bulb (MOB) dataset from the 10x Visium platform [27]. The hematoxylin and eosin(H&E)-stained image in Fig. 2 shows the laminar organization of the coronal MOB. As the 10x Visium platform has locations of void measurement among the lattice arrangement of spots, the padding strategy was performed in the preprocessing step by inserting new unobserved spots for subsequent inference (Supplementary Fig. S2a). STForte was then used to extract latent encodings (ATTR, TOPO, and COMB) from the data. Clustering was conducted using mclust [28] to identify spatial regions. The laminar structure was well recognized with considerable clustering results of STForte (COMB) encoding, while its UMAP visualization had a consistent transition (Fig. 2b,c, Supplementary Fig. S2b, c). Subsequently, we propagated the spatial region identification result from the observed spots to the padding spots using three different encodings. Here, TOPO encoding provided more refined organizations and distinct boundaries for the laminar structure (Fig. 2c, Supplementary Fig. S2d).

Next, we examined some layer-specific marker genes according to the spatial region annotations recognized by STForte. The results showed that they were expressed at relatively distinct levels within their corresponding layers (Fig. 2d,e, Supplementary Fig. S3 and Wilcoxon rank-sum test results for differential expression analysis in Supplementary Table S1), which is concordant with previous reports and validations [29–33]. The padding visualizations (Fig. 2f) markedly enhance the understanding of spatial organization, offering a more consistent and detailed perspective of spatial expression patterns. Additionally, we evaluated the spatial region identification of the dataset using STAGATE [11] and Correspondence Analysis (CA) [34]. We then compared these approaches with our method (Supplementary Fig. S2e–g). STForte achieved the highest silhouette coefficient (SC), while STAGATE recorded the highest C-H index.

### Analyzing spatial cell-resolution MOB data from Stereo-seq using STForte

Next, we examined the capability of STForte to analyze SRT data at cellular resolution. We applied STForte to the MOB dataset obtained from the Stereo-seq platform, which utilizes cell segmentation and aggregated UMI counts to acquire cell-binned SRT data [20]. Figure 2g shows the DAPI-stained image with manual annotation of the laminar structure of the MOB. In this dataset, certain spots exhibit poor expression measurement quality (Supplementary Fig. S4a). STForte allows for the retention of low-quality spots by inferring their biological patterns based on the spatial context of neighboring cells. We conducted spatial region identification using STForte (COMB) encoding and compared the results with those from STAGATE and CA (Fig. 2h). STForte’s results revealed the most consistent spatial regions, accurately distinguishing the laminar structure of the MOB. We further explored the expression of layer-specific genes [29, 31–33, 35–37]. The results showed that these genes were distinctly expressed within their corresponding regions identified by STForte (Supplementary Fig. S4b, c; Wilcoxon rank-sum test results for differential expression analysis in Supplementary Table S2), which is consistent with previous findings. In contrast, although STAGATE provided accurate annotations, the consistency between regions was comparatively lower. As for CA, which does not consider spatial information, the overall regional distribution appeared to be more scattered. In the UMAP visualizations of the three methods (Fig. 2i), STForte presented a continuous transition across different MOB regions, which was not observed in the UMAP visualizations of STAGATE and CA. We compared the clustering performance metrics of the latent space across different methods (Fig. 2j), in which STForte performed better in terms of both the SC and C-H index. Furthermore, STForte accurately recovered the biological patterns of masked spots, consistently assigning them to their neighboring spatial regions (Fig. 2k).

### Exploring TME of prostate adenocarcinoma SRT data with STForte

Leveraging SRT data for cancer analysis presents significant challenges. The invasive growth of tumors, which penetrates their original location and infiltrates the surrounding tissues, results in a mixture between the tumor and the normal area. Moreover, tumors are often accompanied by inflammatory responses and immune cell infiltration. These inflammatory regions do not always perfectly align with tumor cell areas, adding further complexity to the spatial distribution of the TME [14–16].

**Figure 3:**
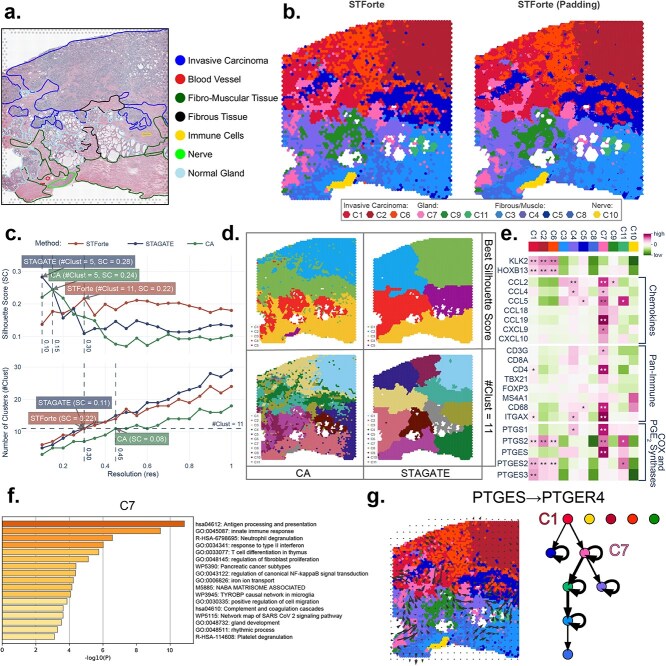
**b**, H&E-stained image with pathological annotations of the 10x Visium prostate adenocarcinoma. **b**, Spatial region identification using STForte ATTR encoding (left) and results from the padding scenario with STForte TOPO encoding (right). **c**, SC of STForte, CA, and STAGATE under the Leiden method at various resolutions. **d**, Spatial visualization shows the Leiden results from STAGATE and CA at their respective best SCs or when #Clust=11. **e**, Heatmap depicting the mean expression levels of genes associated with cancer, immune response, inflammation, and PGE${_{2}}$ synthesis across distinct spatial regions. The statistical significance of higher relative expression within each region was assessed using one-sided Wilcoxon rank-sum tests. The stars denote significance levels: *: *P* <0.05; **: *P* <0.001. **f**, Significant biological pathways associated with region C7 based on the differentially expressed genes. **g**, Spatial interaction between *PTGES* and *PTGER4* genes based on STForte-identified regions and COMMOT analysis.

We analyzed the prostate adenocarcinoma data from the 10x Visium platform using STForte. Figure 3a shows the H&E-stained image and pathological annotations. We employed STForte and performed clustering using the Leiden [38] method, varying its resolution to compare the SCs (Supplementary Fig. S5a). We selected ATTR (Supplementary Fig. S5b, c) as the primary encoding, and the spatial regions are shown in Fig. 3b-left. In addition, padding provided consistent results and a fine-grained view (Fig. 3b-right, Supplementary Fig. S5d, e). We also compared the STForte results along with STAGATE and CA. Figure 3c shows the SC and number of clusters at different resolutions of the Leiden method. STAGATE and CA had higher SC at lower resolutions, but lower SC when STForte reached its maximum or at the same number of clusters. CA could not distinguish the entire invasive carcinoma area from the tissue structures at its highest SC. With the same number of clusters as STForte, CA was able to reflect the TME composition but had a lower SC (Fig. 3d and Supplementary Fig. S5f). STAGATE failed to describe the TME or distinguish tumors from other tissues.

Furthermore, we investigated the expression of cancer- or immune-related genes across different spatial regions identified by STForte (Fig. 3e, Supplementary Fig. S6). *KLK2* and *HOXB13* have been reported to be strongly associated with the proliferation and metastasis of prostate cancer cells [39–41], and their expression levels were elevated in the invasive carcinoma regions. Chemokines [42, 43] and pan-immune genes [44–48] were found to be relatively enriched in C7. Hence, we speculated that C7, identified by STForte, is a potential tertiary lymphoid structure (TLS) [49, 50] that exhibits intense cancer-related immune inflammatory responses. Pathway analysis of differentially expressed genes also suggested that in C7 (Fig. 3f and Supplementary Fig. S7), the significant pathways were related to immunity or cell migration, further indicating the functional association with the TLS region.

Based on these results, we focused on the prostaglandin-related immunological processes in cancer [51]. *PTGS1*, the gene encoding COX-1, was highly expressed in C7, whereas *PTGS2*, the gene encoding COX-2, was highly expressed in both immune and cancerous regions (Fig. 3e and Supplementary Fig. S6). *PTGS1* is a housekeeping gene that maintains basic prostaglandin levels [52], whereas Prostaglandin E2 (PGE$ {_{2}}$) induced by *PTGS2* may lead to tumor-promoting inflammatory responses [53]. Furthermore, mPGES-1, mPGES-2, and cPGES are enzymes involved in PGE$_{2}$ synthesis. It can be observed that *PTGES* (mPGES-1) was found highly expressed in the C7, whereas *PTGES2* (mPGES) and *PTGES3* (cPGES) had relatively higher expression in the cancerous regions. Previous studies have shown that mPGES-1 is an inducible isomerase related to rapid pro-inflammation, whereas mPGES-2 and cPGES are constitutively expressed to sustain tumor survival [54, 55]. Additionally, we analyzed the spatial interactions of prostaglandin-related senders/receivers recorded in CellPhoneDB [56] based on STForte’s results and COMMOT [57] (Fig. 3g, Supplementary Fig. S8, 9), in which C7 plays an important mediator. Activation of these PGE$_{2}$ receivers has been shown to promote tumor spread through inflammation in various cancers [58].

### Spatial smoothing control and fine-grained gene padding with oral squamous cell cancer

**Figure 4:**
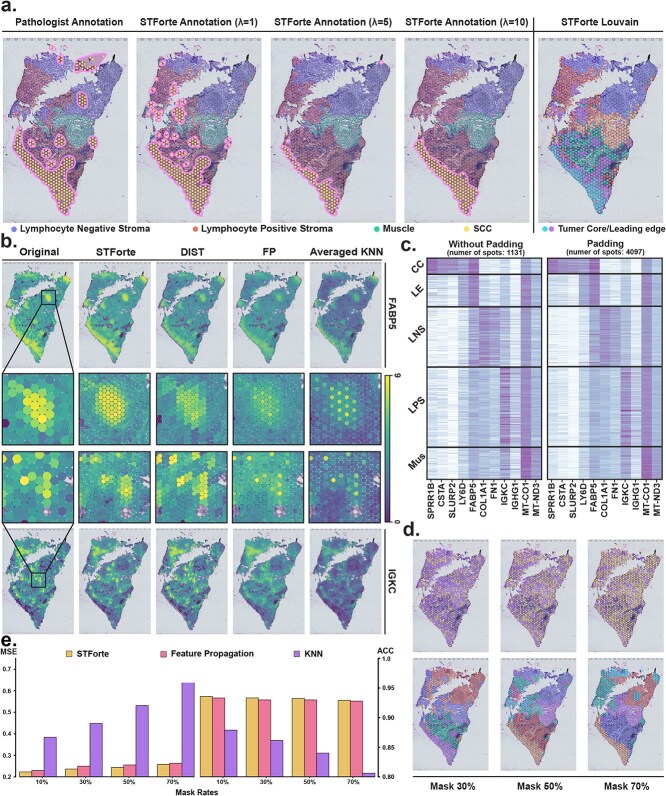
Example results of cancer edge identification with adjustment of $\lambda _{\mathrm{cross}}$ and fine-grained gene expression padding. **a**, The manually combined cluster labels (ATTR encoding) under different $\lambda _{\mathrm{cross}}$ values (left 1–4). Original Louvain clusters when $\lambda _{\mathrm{cross}}=1$ (right 1). **b**, Visualization of the comparison between padded gene expression of STForte and other methods for cancer leading edge (LE) marker *FABP5* and lymphocyte positive stroma (LPS) marker *IGKC*. **c**, Gene expression profiles of top highly variable genes in different tissue areas before and after gene padding. **d**, Clustering results of spatial domain padding (bottom) with randomly masked spots (top). **e**, Robustness of gene padding with randomly masked spots.

STForte aims to address the over-smoothing issue by fine-tuning the cross-reconstruction factor $\lambda _{\mathrm{cross}}$ (see Methods section). We performed spatial domain identification on 12 oral squamous cell carcinoma (OSCC) samples from the 10x Visium platform with STForte to substantiate its applicability [59]. The ATTR encodings, generated under different $\lambda _{\mathrm{cross}}$ values, were clustered using the Louvain [60] algorithm at a resolution of 0.4, followed by a manual consolidation of clusters based on histological images (Supplementary Fig. S10, 11). An example result is presented in Fig. 4a. Notably, when $\lambda _{\mathrm{cross}}$ was set to 1, STForte exhibited the potential to distinguish discrete sporadic cancer areas that were highly consistent with the pathologist’s annotations (Fig. 4a left 1–4). A small $\lambda _{\mathrm{cross}}$ could encourage STForte to encode fine-grained, heterogeneous identities within the spatial domains. Besides, the original Louvain clusters tended to provide suspicious separation of cancer cores and leading edges (Fig. 4a right 1), where these findings are also corroborated by the original findings [59].

STForte also excels in refining padding tasks at the gene expression level. This was accomplished through forecasting using XGBoost regression [61], leveraging the latent encodings generated by STForte. The expression patterns of highly variable genes, once padded by STForte, aligned with their original low-resolution counterparts across various tissue regions, as illustrated in Fig. 4b,c. To demonstrate the advantages of STForte, we further compared its performance with another image-free expression enhancement method, DIST [26], and two baseline methods: FP and averaged KNN. STForte can accurately deduce local expression profiles, even when only sparse high-expression spots were detected. Oppositely, the other methods produce noticeable artifacts. Besides, although STForte failed to fully identify cancer leading edges (Fig. 4a right panel) that are highlighted in the upper half of 4b, it precisely imputed the marker gene expression profile within that region. However, the gene padding results based on STForte’s encodings accurately recovered the expression patterns of its marker genes. This demonstrates the quality of STForte’s encodings and highlights the necessity for a reasonable selection of clustering methods.

To further assess the capabilities of STForte, we randomly masked spots within the original resolution and subsequently compared the recovery outcomes using STForte with those using FP and averaged KNN. DIST was not included here since it cannot infer the expression profiles of irregularly shaped spots. Figure 4d highlights the masked spots and presents the clustering results generated by STForte. The findings indicate that STForte can accurately recover spatial details when the masked rate is below 30%. Even with a masked rate exceeding 70%, STForte still recovered the spatial domain information with acceptable accuracy. This demonstrates the robustness and inferential ability of STForte. As the masked rate increased, the spatial information recovered became less detailed, which aligned with the observed decreasing trend in mean squared error (MSE) and accuracy (ACC) scores for all methods, as depicted in Fig. 4e. Notably, STForte achieved the lowest MSE and highest ACC score among the evaluated methods.

### Employing STForte for analysis of human dorsal lateral prefrontal cortex data

**Figure 5:**
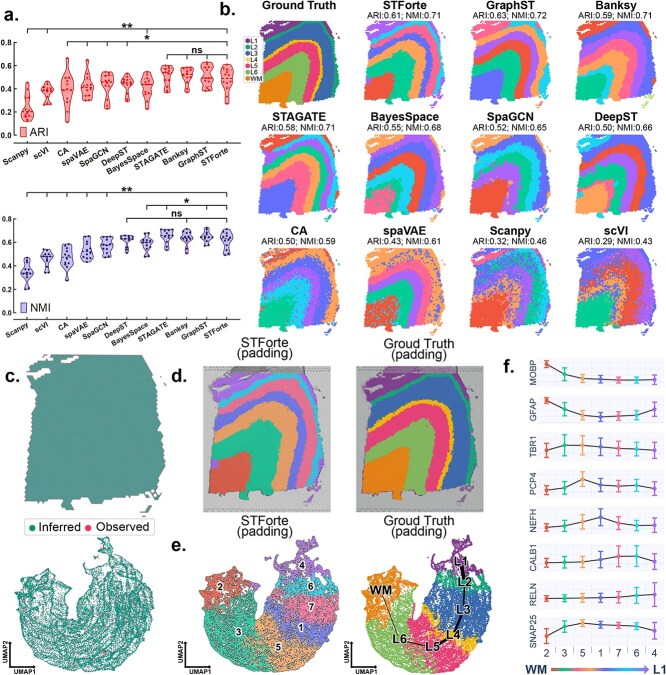
Investigations on the 10x Visium DLPFC dataset. **a**, Violin plot of spatial region identification performance across the 12 slices of the dataset using different approaches, quantified by the ARI and NMI metrics. The dashed lines represent the quartiles and median across 12 points. The asterisks denote significance levels: *: *P*$\leq $ 0.1; **: *P*$\leq $ 0.01; ns: *P* > 0.1. **b**, Spatial regions and performance metrics obtained by different approaches on slice No. 151673. **c**, Spatial (top) and UMAP visualization based on the COMB encoding (bottom) show the spot instances, including observed spots (Observed) and unmeasured intervals (Inferred), obtained by STForte’s padding strategy for slice No. 151673. **d**, Spatial visualizations of the propagated annotations of the padding scenario using spatial identification results (left) or manual annotation (right) based on TOPO encoding. **e**, UMAP visualization and trajectory analysis based on the COMB encoding for the propagated annotations. **f**, Trend plots display averaged expression levels of layer-specific marker genes based on the spatial region identified by STForte, ordered from WM to L1, with error bars showing standard deviations.

Next, we performed a systematic analysis using STForte on the human dorsal lateral prefrontal cortex (DLPFC) data generated by the 10x Visium platform. Clustering was conducted on the three latent encodings produced by STForte across 12 slices to obtain spatial regions. Supplementary Fig. S12a and Supplementary Table S3 show the clustering performance measured by Adjusted Rand Index (ARI) and Normalized Mutual Information (NMI), and the performance comparisons by Wilcoxon signed-rank test. It can be observed that both ATTR and COMB encodings performed well in spatial region identification. We selected COMB encoding as the primary encoding in this investigation. Figure 5a and Supplementary Fig. S13a illustrate the ARI, NMI, and spatial regions across 12 slices for STForte and other methods. STAGATE, GraphST, Banksy, and STForte exhibited comparable performance (Wilcoxon signed-rank test results for performance comparison are provided in Supplementary Table S4). Methods that incorporate spatial information, including BayesSpace, DeepST, SpaGCN, spaVAE, and the aforementioned approaches, exhibited significant improvements in ARI and NMI compared with nonspatial methods. Figure 5b and Supplementary Fig. S12b illustrate the spatial domain identification results from the different methods and STForte encodings on slice No. 151673, respectively. Nearly all spatial information-based methods could identify consistent and structured spatial regions within the DLPFC tissue. We also conducted an ablation study of STForte using 12 slices, which confirmed the necessity of all key modules in the framework (Supplementary Fig. S13b). We also conducted a trajectory analysis based on PAGA using latent encodings provided by different approaches (Supplementary Fig. S12c). The latent encodings of STForte presented fluent transitions from the white matter (WM) to Layer 1 (L1).

Moreover, using the padding strategy of STForte (Fig. 5c, Supplementary Fig. S12d), we could obtain more fine-grained spatial regions (Fig. 5d) to elucidate the DLPFC structure. Trajectory analysis incorporating the imputed properties in the padding scenario also demonstrated the continuity of the structure from WM to L1 (Fig. 5e). We designed an RGB analysis to visualize the differences between the three encodings when applying the padding strategy to this dataset (Supplementary Fig. S12e). Each encoding was processed using UMAP to reduce its dimensionality to three, followed by the standardization of each dimension and mapping to the RGB space. It can be observed that the encodings obtained through feature propagation using ATTR (ATTR_FP) are not as smooth as TOPO or COMB encodings in space, which is consistent with the findings from other 10x Visium-based analyses discussed in the previous sections.

We further analyzed the expression patterns of layer-specific marker genes. Based on the spatial regions identified by STForte, these marker genes displayed distinct changes in expression levels, corresponding to the genuine structure of the human DLPFC (Fig. 5f) [62–70]. Furthermore, by leveraging the padding strategy, STForte can impute the gene expression levels for unobserved locations, yielding more fine-grained measurements of expression profiles (Supplementary Fig. S14).

Moreover, we utilized data from DLPFC No.151563-151676 for analysis in a multi-slice integration scenario. Specifically, we first aligned the spatial coordinates of different slices using PASTE2 [71]. Based on the aligned coordinates, STForte was employed to construct an integrated latent space in a multi-slice perspective. The spatial region identification results in the multi-slice context are shown in Supplementary Fig. S15a. Notably, STForte yielded continuous and consistent spatial regions in the multi-slice view. Additionally, STForte enabled more fine-grained spatial regions by adopting a padding strategy. Comparing the results with other methods, STForte demonstrated the highest averaged ARI and NMI in multi-slice integration. Moreover, STForte achieved the second-highest iLISI [72] (Supplementary Fig. S15b), surpassed only by GraphST. The UMAP visualization also illustrated that STForte’s latent space could provide clear region delineation and considerable cross-slice integration (Supplementary Fig. S15c).

### Elucidating anatomical structures in Xenium mouse brain data using STForte

**Figure 6:**
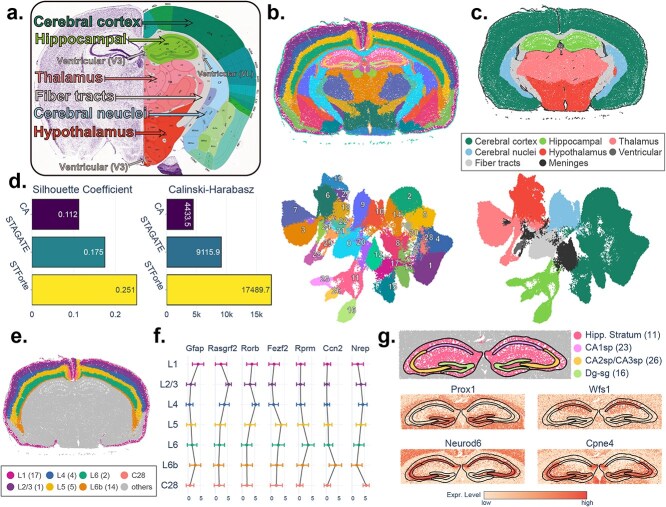
Analysis of Xenium mouse coronal brain data using STForte. **a**, Anatomical annotation of a coronal section of the mouse brain. Adapted from the Allen Brain Atlas. **b**, Spatial region identification based on STForte ATTR encoding on the Xenium mouse coronal brain dataset (top) and corresponding UMAP visualization (bottom). **c**, Spatial (top) and UMAP (bottom) visualizations of anatomical parcellation based on the summarized STForte results. **d**, Comparison of clustering performance metrics for different methods on this dataset. **e**, Visualization of the spatial regions identified by STForte in the Isocortex. **f**, Average expression levels of layer-specific marker genes in the outer cortical layers are shown with error bars representing the standard deviation. **g**, Hippocampal regions identified by STForte and the spatial expression patterns of relevant marker genes.

In this section, STForte was applied to the 10x Xenium mouse coronal brain dataset, which contains *in situ* spatial measurements of over 130 000 cells in the entire mouse brain. Fig. 6a illustrates the referenced anatomical structure of the mouse coronal brain from the Allen Atlas [73]. The COMB encoding of STForte was employed for downstream spatial region identification via clustering. It can be observed that different anatomical regions of the mouse brain were clearly identified, while the UMAP embedding space well depicted the functional distinction among cells within the entire brain (Fig. 6b). The clustering results were further annotated according to their anatomical characteristics, as summarized in Fig. 6c and Supplementary Table S5. The annotated domains aligned well with the reference anatomical structures shown in Fig. 6a. We also performed STAGATE and CA workflows and compared them with STForte. STForte achieved higher SC and the C-H index than the other two methods (Fig. 6d, Supplementary Fig. S16).

Furthermore, we inspected the expression patterns of known marker genes to evaluate whether STForte accurately delineated subregions in different anatomical domains. Figure 6e,f illustrates the regions of the Isocortex identified by STForte and the expression levels of different layer-specific genes [74–82] (Wilcoxon rank-sum test results are provided in Supplementary Table S6). These layer-specific expression patterns highlight the distinct genetic profiles of each cortical layer, demonstrating the ability of STForte to accurately identify these layers. Moreover, expression patterns in the hippocampus [83–86] are also highly concordant with the regions identified by STForte (Fig. 6g and Supplementary Table S7). These results demonstrate that STForte can perform precise anatomical region identification of complex brain structures at single-cell resolution.

## Discussion and conclusion

In this study, we present STForte, a representation learning and spatial enhancement method for SRT data analysis. STForte incorporates graph structures during encoding, assuming that neighboring spots share similar expression profiles to preserve spatial correlations. However, the excessive preservation of spatial graphs can be counterproductive, especially for nonconsecutive spatial domains. To address this, STForte employs a heuristic cross-reconstruction training strategy, balancing attribute and topology encoders to avoid overemphasizing spatial homogeneity. This approach effectively characterizes the heterogeneous tissues, such as the TME. Experimental results demonstrate STForte’s effectiveness and controllability in integrating spatial and attribute information.

SRT data exhibit significant diversity and heterogeneity, and are influenced by factors such as disease type, sample area, and sequencing technologies. STForte is designed to adapt to data from various SRT technologies and analysis scenarios through simple and flexible fitting procedures. For user guidance, we provide a recommended recipe in Supplementary Fig. S18 and detailed parameter selections for the experiments in Supplementary Table S8.

To reduce GPU memory usage during STForte training, we introduced a partial adjacency reconstruction trick. Instead of reconstructing the entire adjacency matrix, this technique recovers a random subset at each epoch, significantly optimizing memory efficiency. This allows STForte to process large single-cell resolution datasets with 100 000 to 500 000 cells/spots on a single 24GB GPU (Supplementary Fig. S19). Although STForte’s training time is longer than that of GNN-based embedding methods without spatial resolution enhancement, it remains practical and efficient (Supplementary Table S9).

In conclusion, developing a representation method adaptable to all SRT data remains challenging. This study proposes an improved representation learning framework combined with a flexible spatial enhancement approach, emphasizing the need to address challenges in applying graph-based algorithms to SRT analysis. Although previous studies have introduced techniques to mitigate over-smoothing, they often lack control over spatial-emphasized effects and may lead to information loss. Future research should focus on designing representation learning methods that better balance the complexities of spatial graph structures and gene expression profiles while achieving superior efficiency and performance.

## Methods

### Problem statement

STForte constructs a graph $\mathcal{G} = (\mathcal{V}, A, X)$ from the spatial coordinates and expression matrix of the SRT data. Specifically, $\mathcal{V} = \{v_{1}, v_{2}, \cdots , v_{N}\}$ denotes the node set that represents the total $N$ spots/cells. Spatial coordinates are used to establish the spatial topology of different spots/cells, denoted as an adjacency matrix $A \in \{0, 1\}^{N \times N}$, which is constructed using either a distance-based neighboring strategy or a KNN strategy, determined by whether the SRT protocol is formulated with a regular lattice (see Supplementary Fig. S1a and Supplementary Note S1 for more details). Expression profiles, denoted by $X \in \mathbb{R}^{N \times M}$, can be either a matrix of gene expression values or results from a nonspatial dimensionality reduction approach (e.g. PCA).

STForte is designed to handle unobserved locations without given expression profiles. To this end, the node set $\mathcal{V}$ consists of two subsets: $\mathcal{V}^{o}=\{v_{1}^{o},v_{2}^{o},\ldots ,v_{N_{o}}^{o}\}$ representing the $N_{o}$ observed spots/cells, and $\mathcal{V}^{u}=\{v_{1}^{u},v_{2}^{u},\ldots ,v_{N_{u}}^{u}\}$ representing the $N_{u}$ unobserved spots/cells. Subsequently, the adjacency matrix and expression profiles corresponding to the observed spots/cells are represented as $A^{o} \in \mathbb{R}^{N_{o} \times N_{o}}$ and $X^{o} \in \mathbb{R}^{N_{o} \times M}$, respectively. The expression profiles of unobserved locations, denoted by $X^{u} \in \mathbb{R}^{N_{u} \times M}$, are unavailable.

The objective of the STForte architecture is to extract graph information ($\mathcal{G}$) while recovering node entities of unobserved locations ($\mathcal{V}^{u}$). Besides, the STForte model aims to comprehensively consider the spatial topology ($A$) and expression profile characteristics ($X$) to provide a contextual description of SRT data. Inspired by Chen *et al*. [87], STForte employs a pairwise autoencoder to derive $l$-dimensional latent-space representations that encompass spatial topology and expression-specific information.

### Latent encoding framework

The latent encodings of SRT data are obtained through a pairwise GAE (Supplementary Fig. S1b), where gene expression profiles and spatial topology information are processed by two distinct encoding structures:

(i) The attribute encoder, a multilayer perceptron (MLP) $f_{\psi _{X}}: \mathbb{R}^{\cdot \times M}\mapsto \mathbb{R}^{\cdot \times l}$, maps expression profiles to $l$-dimensional latent variables, with $\psi _{X}$ denoting the attribute encoder parameters.
(ii) The topology encoder is represented as $f_{\psi _{A}}: \mathbb{R}^{\cdot \times M} \times \mathbb{R}^{\cdot \times \cdot }\mapsto \mathbb{R}^{\cdot \times l}$, which maps the topology information of the graph converted from SRT data to $l$-dimensional latent variables through a Pathfinder Discovery Network [88] (Supplementary Note S2), where $\psi _{A}$ is the topology encoding parameters.

Subsequently, decoders are designed to handle the restoration from the latent-space representation to the original information:

(i) The attribute decoder part $f_{\theta _{X}}: \mathbb{R}^{\cdot \times l}\mapsto \mathbb{R}^{\cdot \times M}$ is an MLP that restores expression profiles from the latent variables, where $\theta _{X}$ indicates the corresponding parameters.
(ii) The topology decoder is defined as $f_{\theta _{A}}: \mathbb{R}^{\cdot \times l}\mapsto \mathbb{R}^{\cdot \times \cdot }$, which is an MLP parameterized by $\theta _{A}$ with an inner product operation followed by the last layer. The inner product operation is used to restore the adjacent matrix $\hat{\mathbf{A}} \in \mathbb{R}^{\cdot \times \cdot }$ as $\hat{\mathbf{A}}=\mathrm{sigmoid}(\hat{\mathbf{Z}}\hat{\mathbf{Z}}^{T})$, where $\hat{\mathbf{Z}} \in \mathbb{R}^{\cdot \times l}$ is the output of the topology decoder.

### Match between latent encodings

We denote $Z_{X^{o}} = f_{\psi _{X}}(X^{o}) \in \mathbb{R}^{N^{o} \times l}$ as the attribute latent encoding of observed spots. For topology encodings, feature propagation method [89] is employed, leveraging the spatial topological structure and observed expression values to impute unavailable expression profiles at unobserved locations (Supplementary Note S3), with these imputed profiles denoted as $X^{\mathrm{fp}} \in \mathbb{R}^{N_{u} \times M}$. The topology encoding is computed as $Z_{A} = f_{\psi _{A}}(A, X^\prime ) \in \mathbb{R}^{N \times l}$, where $X^\prime \in \mathbb{R}^{N \times M}$ represents the expression profiles comprising $X^{o}$ and $X^{\mathrm{fp}}$. Consequently, two adaptive approaches are implemented to align the latent spaces of $Z_{X^{o}}$ and $Z_{A}$ in the STForte training strategy.

#### Reconstruction matching

In the vanilla scheme of the autoencoding framework, the reconstruction loss encourages the latent encodings to reproduce the observation [90]. In STForte, we assume that the attributes and topological features of nodes within the graph are intrinsically correlated and can be projected into a shared latent space. Consequently, the reconstruction of attribute information is formulated as follows:


(1) \begin{align*}& \begin{split} \mathcal{L}_{\mathrm{attr}}=& \frac{1}{N_{o}M} \lVert X^{o} - f_{\theta_{X}}(Z_{X^{o}})\rVert^{2}_{F} + \frac{\lambda_{\mathrm{cross}}}{N_{o}M} \lVert X^{o} - f_{\theta_{X}}(Z_{A^{o}})\rVert^{2}_{F} \end{split},\end{align*}

where $\lambda _{\mathrm{cross}}$ denotes the weight factor for the cross-information reconstruction and $Z_{A^{o}} \in \mathbb{R}^{N_{o} \times l}$ represents the topology latent encoding corresponding to the observed spots/cells $\mathcal{V}^{o}$. $\lVert \cdot \rVert _{F}$ denotes the Frobenius norm. The first term in Equation 1 represents the mean square error that measures the self-reconstruction quality of the attribute information, and the second term represents the cross-reconstruction loss used to restore the attribute information from topology latent encoding. Furthermore, the reconstruction of topology information is formulated as follows:


(2) \begin{align*}& \begin{split} \mathcal{L}_{\mathrm{topo}}=& \mathbb{E}_{p(A)} \left[-\log \left(f_{\theta_{A}}(Z_{A})\right)\right] + \lambda_{\mathrm{cross}}\mathbb{E}_{p(A^{o})} \left[-\log \left(f_{\theta_{A}}(Z_{X^{o}})\right)\right] \end{split}\end{align*}

In Equation 2, the first cross-entropy (CE) term measures the quality of self-reconstruction for topology information, whereas the second CE term qualifies the cross-reconstruction of recovering the node neighboring relations from the observed attribute latent encoding. Subsequently, the overall reconstruction loss is formulated as follows:


(3) \begin{align*}& \begin{split} \mathcal{L}_{\mathrm{recon}}=& \mathcal{L}_{\mathrm{attr}} + \mathcal{L}_{\mathrm{topo}} \end{split}\end{align*}

The objective of the reconstruction matching process is achieved by minimizing $\mathcal{L}_{\mathrm{recon}}$ with respect to the parameters $\psi _{X}, \psi _{A}, \theta _{X}, \theta _{A}$ during training. This allows latent encodings to learn condensed information from the original data, whereas the cross-reconstruction process enables information sharing between latent encodings.

#### Adversarial distribution matching

To further impose closeness between the latent encodings of attribute and topology, STForte utilizes an adversarial-based approach [91] to match their latent spaces. We denote $Z^\prime $ as the latent samples drawn from a reference prior distribution $q(Z)$. The optimization refers to the minimax objective [92], which can be defined as follows:


(4) \begin{align*}& \begin{split} \min_{\phi} \max_{\psi_{X}, \psi_{A}} \mathcal{L}_{\mathrm{adv}}=\,& 2 \mathbb{E}_{q(Z)}\left[-\log(\mathcal{D}(Z^\prime))\right] \\ & +\mathbb{E}_{p(Z_{X^{o}})}\left[-\log(1-\mathcal{D}(Z_{X^{o}}))\right]\\ &+\mathbb{E}_{p(Z_{A})}\left[-\log(1-\mathcal{D}(Z_{A}))\right] \end{split},\end{align*}

where $\mathcal{D}$, parameterized by $\phi $, is an MLP-based discriminator shared among different latent encodings. In STForte, the reference distribution is configured as a standard Gaussian distribution. Equation 4 conducts adversarial distribution matching to encourage the manifolds of topology and attribute latent encodings to be close to the reference prior, implicitly imposing the closeness between the attribute and topology features.

### Overall objective

Combining the above loss functions and objectives, the main objective function of the STForte model in the training process is as follows:


(5) \begin{align*}& \min_{\Theta} \max_{\Psi} \mathcal{L} = \mathcal{L}_{\mathrm{recon}} + \lambda_{\mathrm{adv}} \mathcal{L}_{\mathrm{adv}},\end{align*}

where $\Theta ={\theta _{X}, \theta _{A}, \psi _{X}, \psi _{A}, \phi }$ and $\Psi = {\psi _{X}, \psi _{A}}$. The weight factor $\lambda _{\mathrm{adv}}$ is utilized for the trade-off between the reconstruction and adversarial distribution matching.

### Encodings and property propagation

Supplementary Fig. S17 illustrates the schematic descriptions of the STForte encodings. We described the details of encodings, propagation of annotation, and imputation of gene expression levels for unobserved spots in Supplementary Note S4.

### Details on analysis

Details of the analysis of the datasets are provided in Supplementary Note S5.

### Evaluation metrics and compared methods

The evaluation metrics used in this study are detailed in Supplementary Note S6. Descriptions of the methods selected for comparison are provided in Supplementary Note S7.

**Key Points**

- A novel pairwise GAE framework, named STForte, is proposed for spatial transcriptomics analysis, which generates context-specific encodings for spots/cells by considering the information between expression profiles and spatial topology within the latent space.
- STForte can be used to perform spatial enhancement by imputing the properties of unobserved or mismeasured locations through consistency-aware latent space matching strategy. The spatial enhancement results provide more fine-grained and consistent contextual information for downstream analysis.
- STForte can adaptively encode homogeneous or heterogeneous spatial transcriptomic data while preventing spatial over-smoothing by implicitly modulating the integration of spatial context. Experiments were conducted to demonstrate STForte’s superiority in analyzing spatial data in different scenarios, such as revealing anatomical structures or elucidating complex TMEs.

## Supplementary Material

STForte_Supplementary_Data_bbaf174

## Data Availability

All datasets included in this study are available in raw data from their corresponding resources. The 10x Visium mouse olfactory bulb dataset is available from the Gene Expression Omnibus (GEO) repository (accession No.GSM4656181). The Stereo-seq mouse olfactory bulb dataset [8] is accessible on https://github.com/JinmiaoChenLab/SEDR_analyses/tree/master/data. The 10x Visium FFPE prostate adenocarcinoma dataset can be obtained from the 10x dataset repository (https://www.10xgenomics.com/datasets/human-prostate-cancer-adenocarcinoma-with-invasive-carcinoma-ffpe-1-standard-1-3-0). The human OSCC dataset is available from the GEO repository (accession No. GSE208253). The DLPFC dataset can be downloaded from https://research.libd.org/spatialLIBD/. The 10x Xenium dataset can be obtained from the 10x dataset repository (https://www.10xgenomics.com/datasets/fresh-frozen-mouse-brain-for-xenium-explorer-demo-1-standard, Full coronal section).

## Code availability

The STForte software code is implemented by Python and is public available at https://github.com/poncey/STForte.
