# Supplementary material for: STForte: tissue context-specific encoding and consistency-aware spatial imputation for spatially resolved transcriptomics
Source: Brief Bioinform. 2025 Apr 21;26(2):bbaf174. doi: 10.1093/bib/bbaf174 (PMC12009714; doi:10.1093/bib/bbaf174)
Supplement: STForte_Supplementary_Data_bbaf174 [file stforte_supplementary_data_bbaf174.pdf]

Supplementary Figures

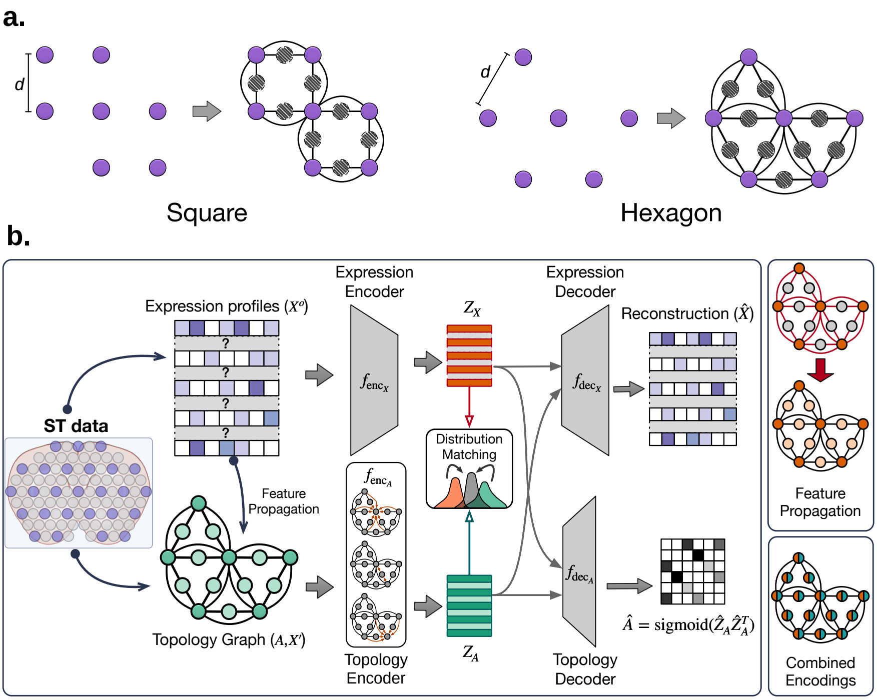

Supplementary Fig. S1 a, Spot padding strategy for intervals of unobserved expressions in regular-lattice SRT data. For square-arranged lattice data, such as Spatial Transcriptomics, or hexagon-arranged data, such as 10x Visium, new spots are inserted based on the center-to-center distance (d). **b,** STForte latent encoding framework. First, expression profiles and spatial topology are obtained from the SRT data. The extracted information is subsequently fed into a pairwise graph autoencoder (GAE), where the expression profiles of observed locations (X^o) and topology graph, including spatial neighbors A and expression profiles with imputed data for unobserved locations thorugh feature propagation X', are separately encoded into their corresponding latent spaces (Z_X and Z_A). The fitting process includes (1) self/cross-reconstruction streams to restore the expression attributes ($\hat{X}$) and adjacent matrix ($\hat{A}$) for spatial-aware dimensional reduction and latent-space alignment, and (2) adversarial distribution matching to further control the latent space of both encondings. Finally, Z_X (ATTR), Z_A (TOPO), and their combined encodings through concatenation (COMB) can be used for diverse downstream analyses.

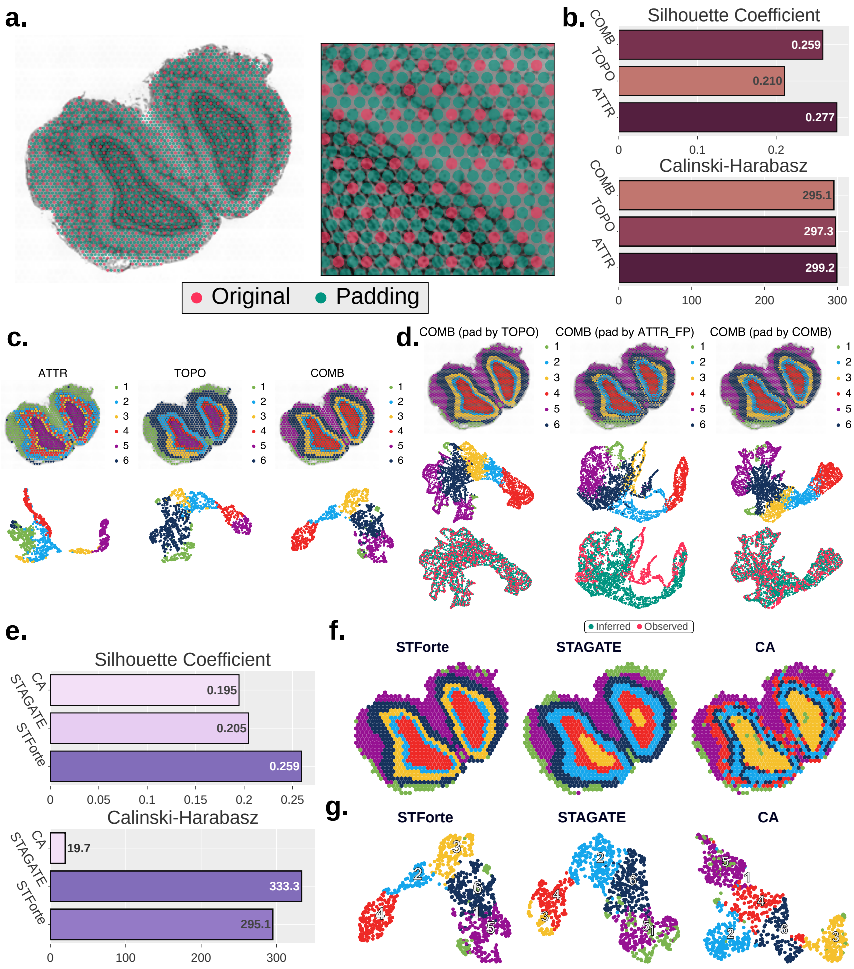

Supplementary Fig. S2 a, Spot instances including well-measured spots (original) and unmeasured intervals generated by STForte's padding strategy. **b,** Comparison of clustering metrics among different STForte encodings, including the Silhouette Coefficient and the Calinski-Harabasz index. **c,** Spatial regions (top) and UMAP visualizations (bottom) of different STForte encodings. **d,** Spatial regions (top), UMAP visualizations based on region annotations (middle), and spot instances (bottom) of different STForte encodings under padding scenario. **e,** Comparison of clustering metrics for different dimensional reduction methods (STForte, STAGATE, and CA). **f,** Spatial regions identified by different methods. **g,** UMAP visualizations obtained by different methods.

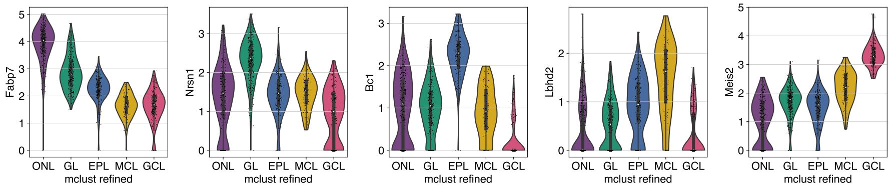

Supplementary Fig. S3 A detailed violin plot shows the expression levels of different layer-specific marker genes across various layers.

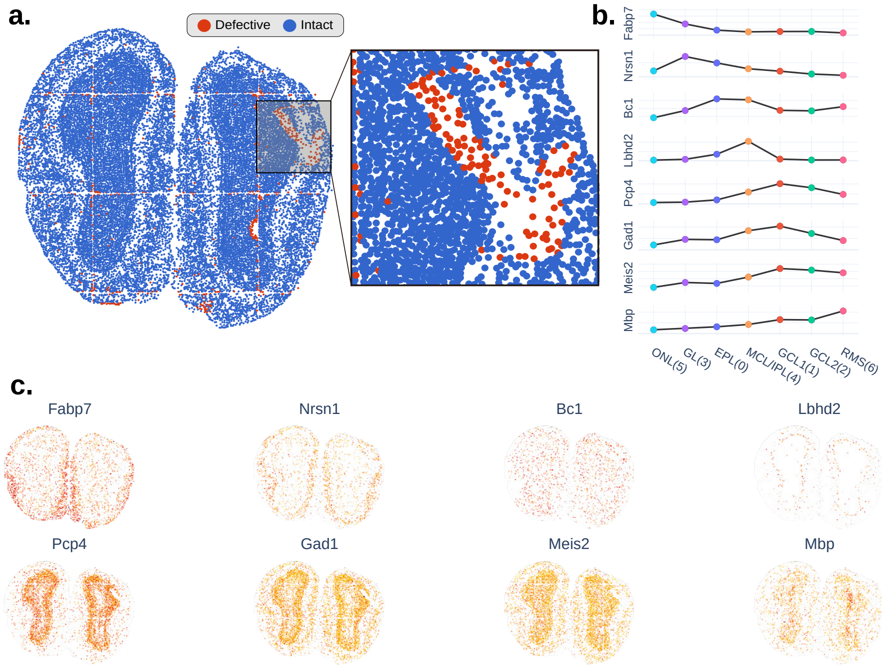

Supplementary Fig. S4 a, Spot instances including well-measured cells (Intact) and low-quality cells (Defective) masked for the STForte process. **b,** The tendency plot shows the mean expression of different genes within different spatial regions. **c,** Visualization of spatial expression levels for the layer-specific genes.

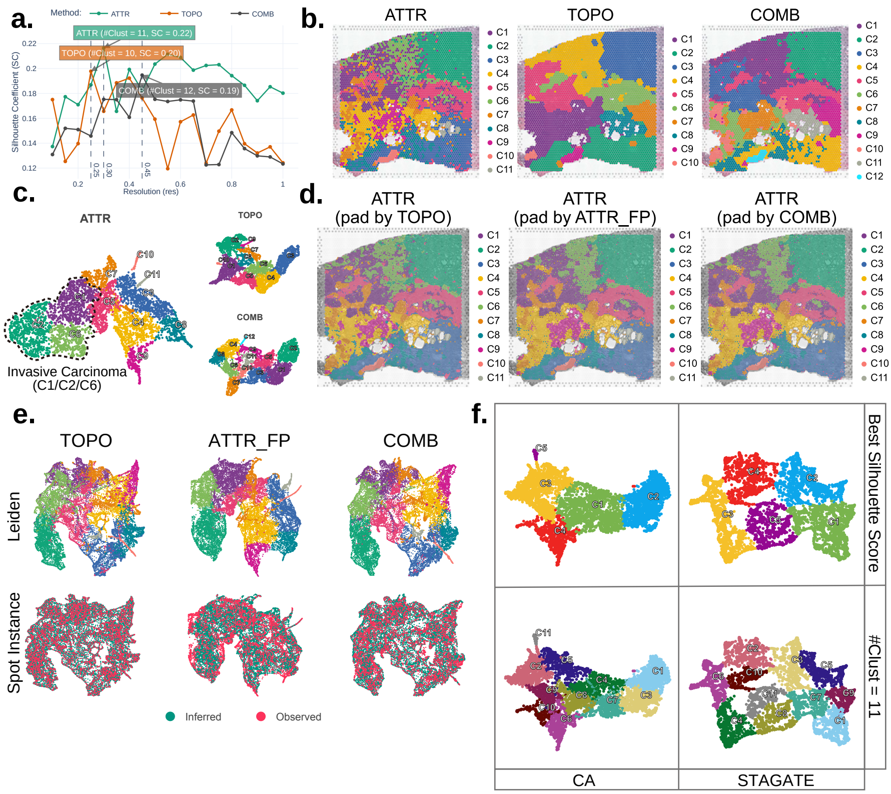

Supplementary Fig. S5 a, Silhouette coefficients (SC) of different STForte encodings under the Leiden method at various resolutions. #Clust indicates the number of clusters. **b,** Spatial regions of different encodings at the best silhouette coefficient under Leiden clustering. **c,** UMAP visualization of different STForte encodings. For ATTR, C1, C2, and C6 correspond to invasive carcinoma. **d,** Spatial regions of different STForte encodings under padding scenario. **e,** UMAP visualizations based on Leiden results and spot instances of different STForte encodings under padding scenario. **f,** UMAP visualization shows the Leiden results from STAGATE and CA at their respective best silhouette coefficients or when #Clust=11.

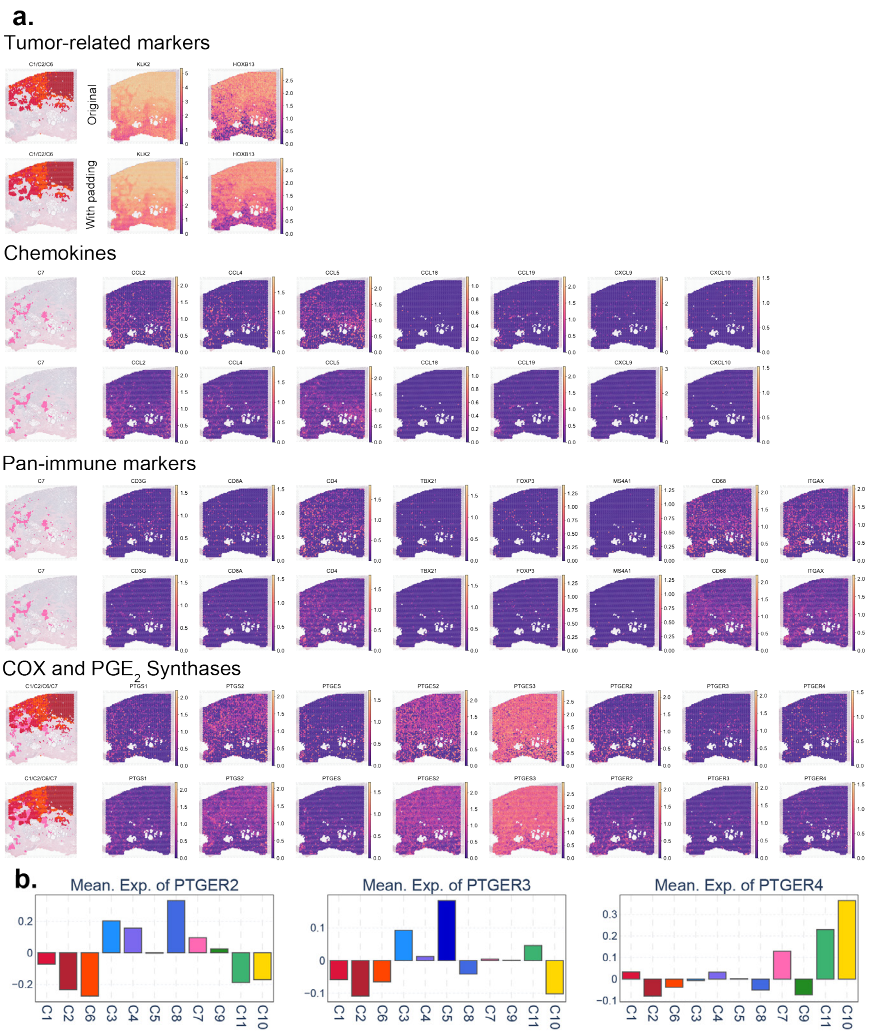

Supplementary Fig. S6 a, Investigated the spatial expression levels of cancer and immune-related genes, including results after STForte padding operation. **b,** Mean expression levels of different PGE_2 receptors.

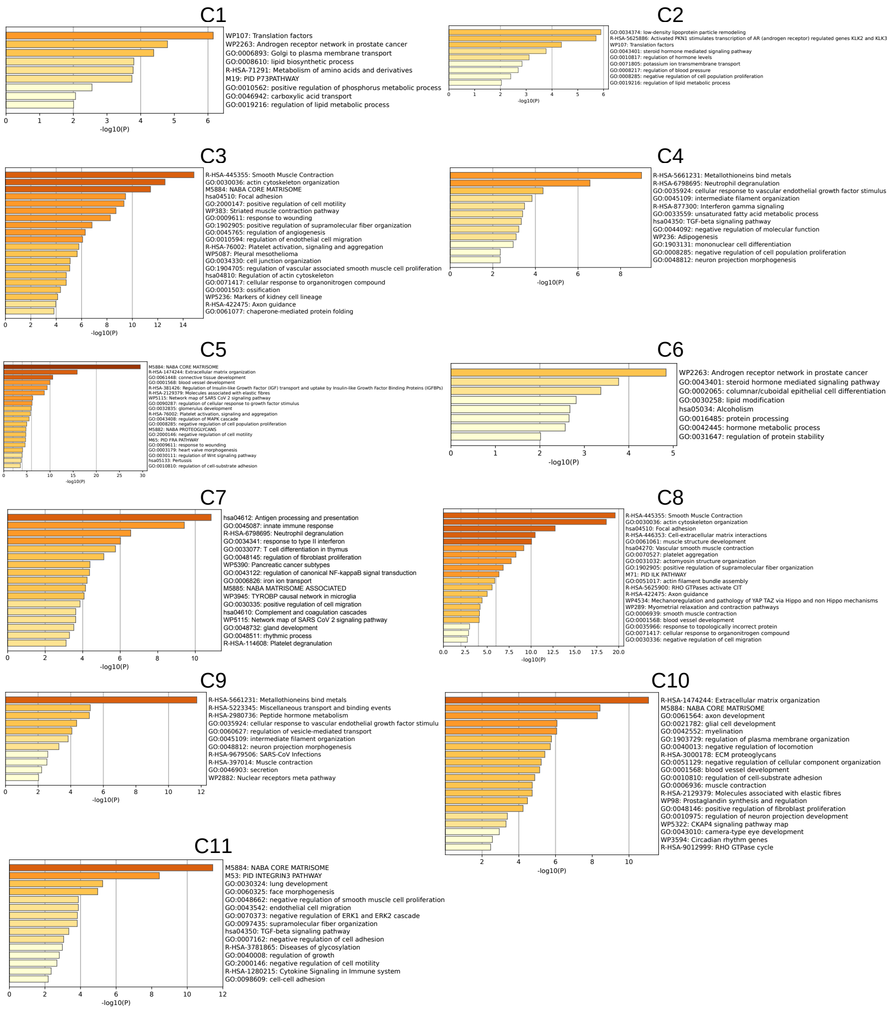

Supplementary Fig. S7 Functional pathway analysis based on top differentially expressed genes specific to different spatial regions identified by STForte.

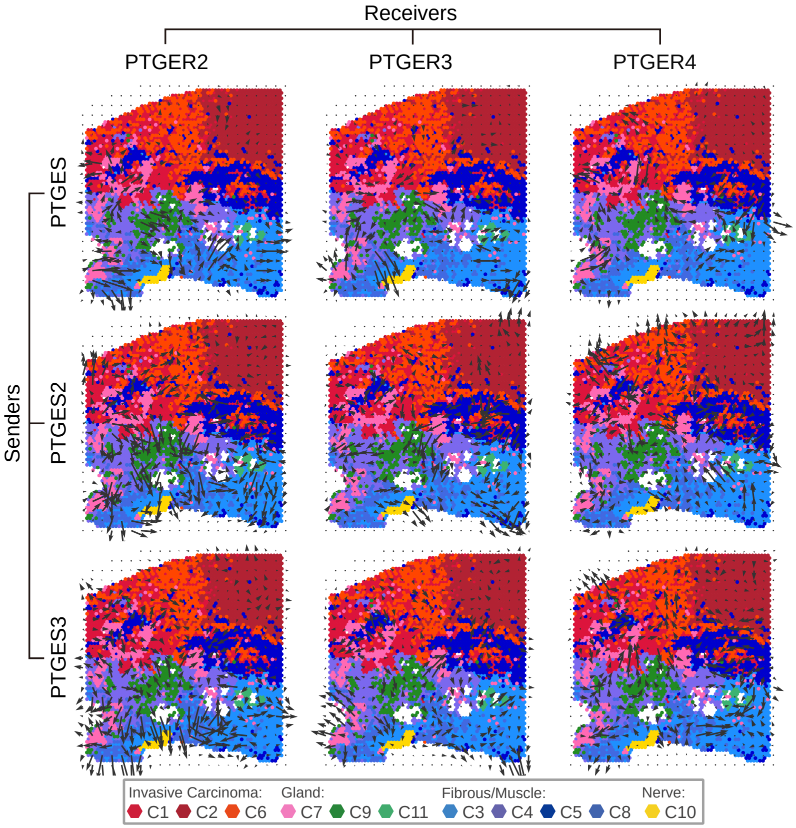

Supplementary Fig. S8 The spatial interaction of relevant genes in the PGE_2 pathway, obtained by analyzing the results of spatial region identification using STForte in combination with COMMOT.

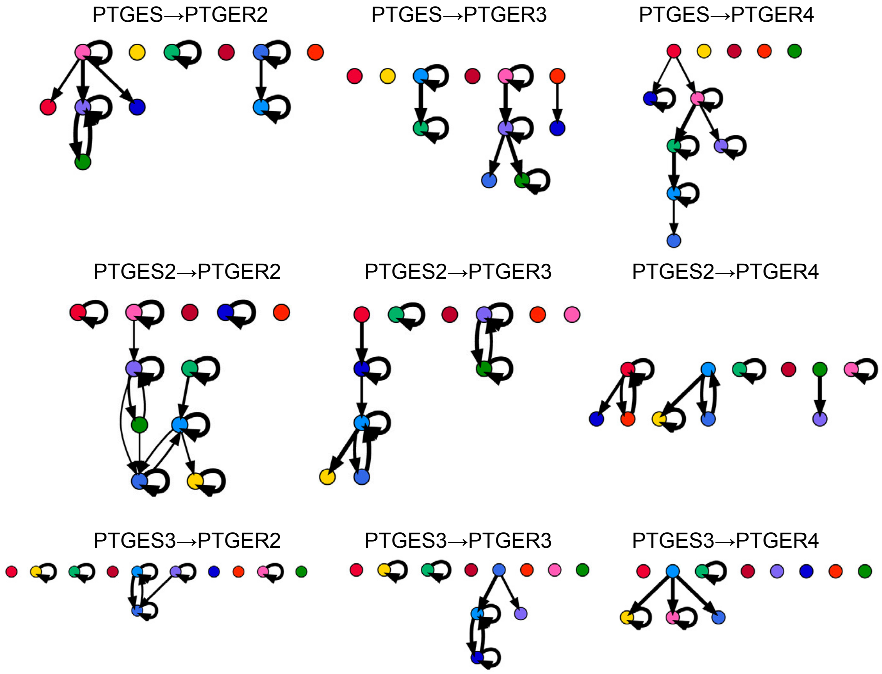

Supplementary Fig. S9 The interaction among spatial regions of relevant genes in the PGE_2 pathway, obtained by analyzing the results of spatial region identification using STForte in combination with COMMOT.

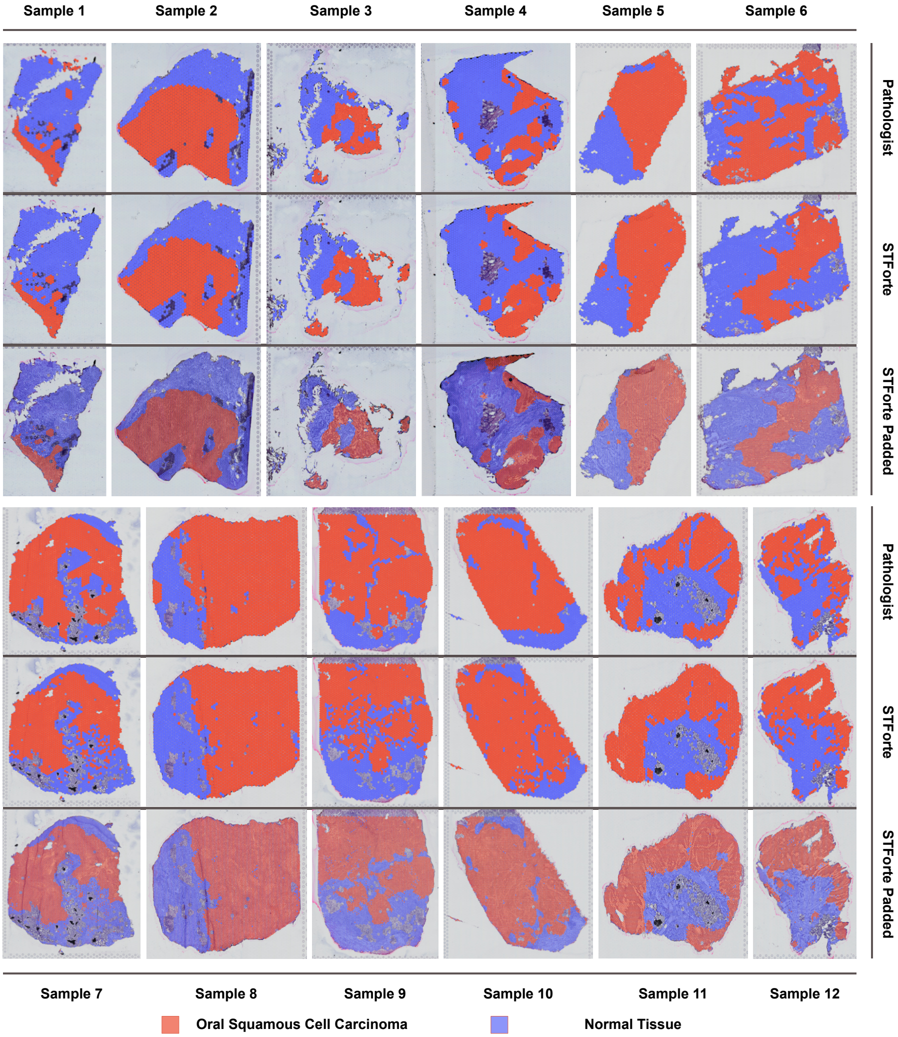

Supplementary Fig. S10 Cancer region identification results of STForte for all 12 OSCC sections with padding. The SCC region was annotated through the Louvain algorithm with a resolution of 0.4 followed by manual cluster integration.

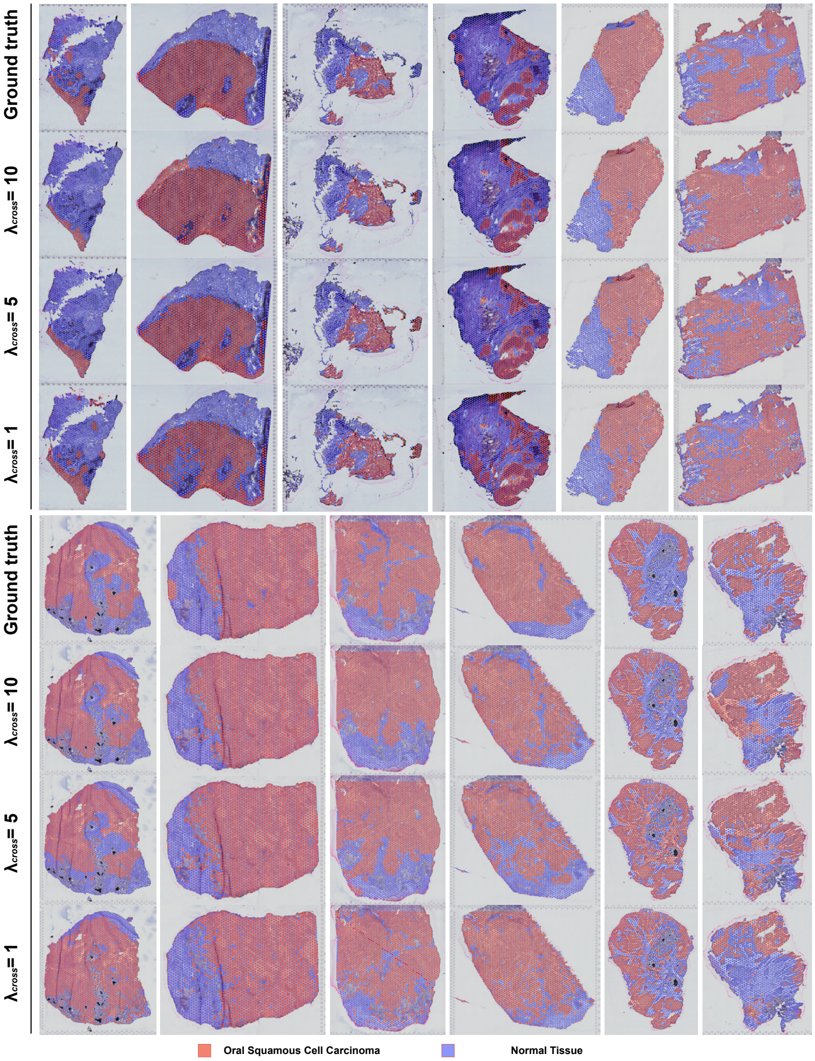

Supplementary Fig. S11 Cancer region identification results of STForte under different settings of λ_{cross}.

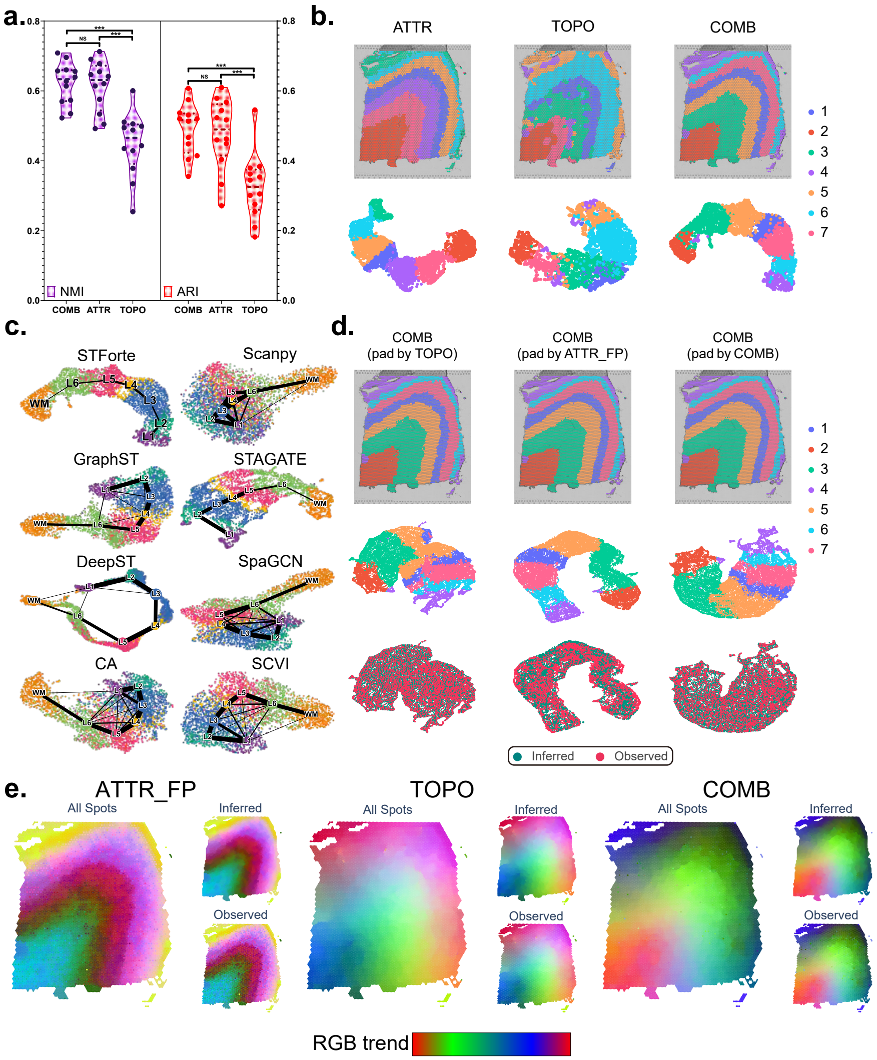

Supplementary Fig. S12 a, Violin plot of spatial region identification performance across the 12 slices of the dataset using different STForte encodings, quantified by the ARI and NMI metrics. The dashed lines represent the quartiles and median across 12 points. **b,** Identified spatial regions (top) and UMAP visualizations (bottom) obtained from different STForte encodings. **c,** Trajectories based on different latent embedding approaches incorporated with PAGA. **d,** Spatial regions (top), UMAP visualizations based on region annotations (middle), and spot instances (bottom) of different STForte encodings under padding scenario. **e,** RGB analysis depicting the identical spatial patterns and fluency of latent spaces from different STForte encodings within slice No. 151673.

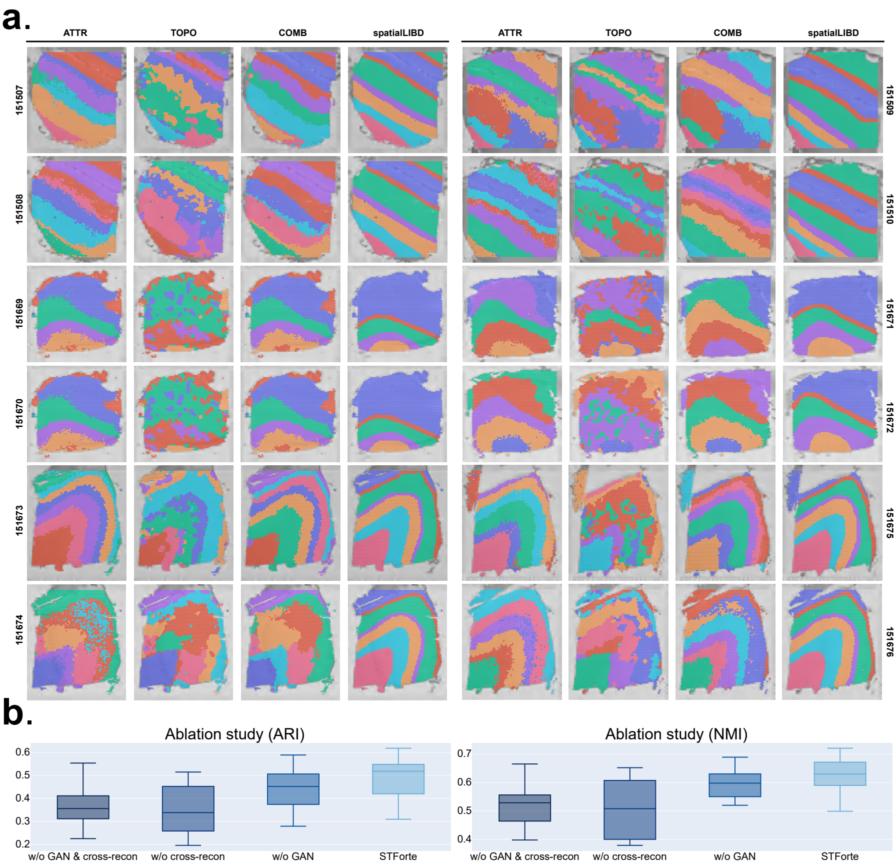

Supplementary Fig. S13 a, Clustering results of all 12 sections of DLPFC. **b,** Ablation study for STForte. Comparing results without both adversarial distribution matching (GAN) and cross-reconstruction versus removing only the GAN module shows significant improvement in median clustering metrics. Using GAN alone did not improve performance over the baseline architecture. However, combining GAN with cross-reconstruction significantly improved median clustering performance, as distribution matching between attribute and topological embeddings enables encoders to learn from each other, facilitating the cross-reconstruction process.

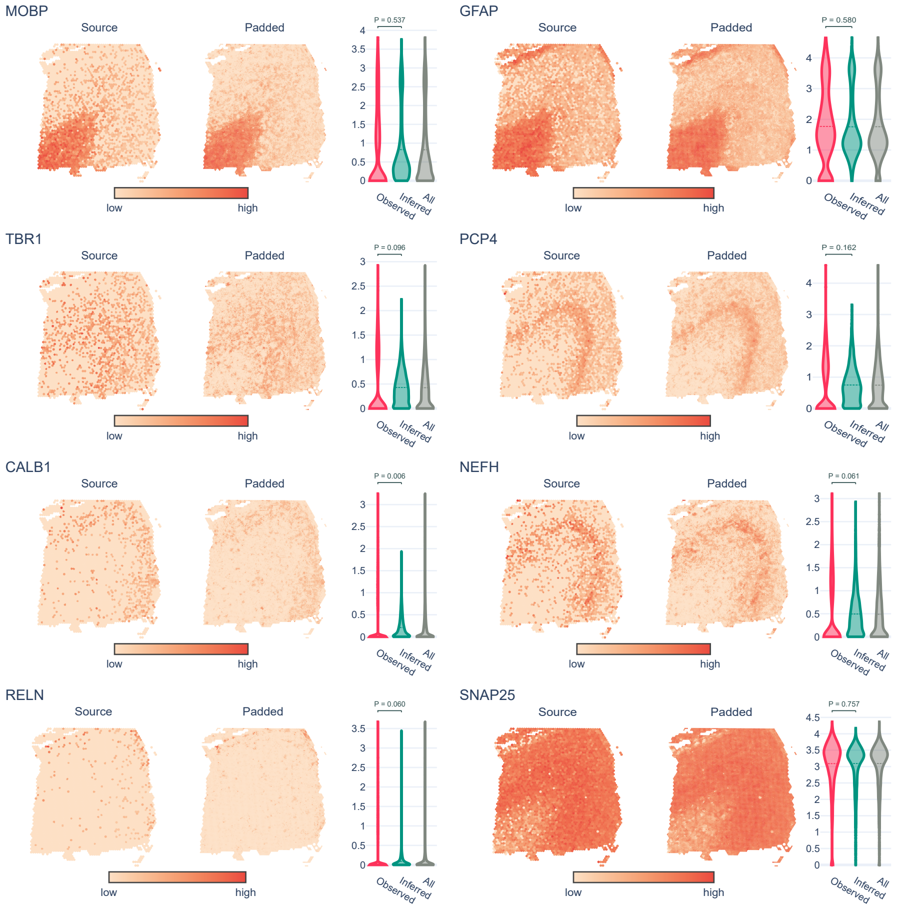

Supplementary Fig. S14 Spatial gene expression levels of investigated layer-specific marker genes in the originally observed spots (source) and under the padding scenario. Violin plots depict the expression levels in observed spots, unobserved (inferred) spots, or all spots. P-values were obtained through t-tests.

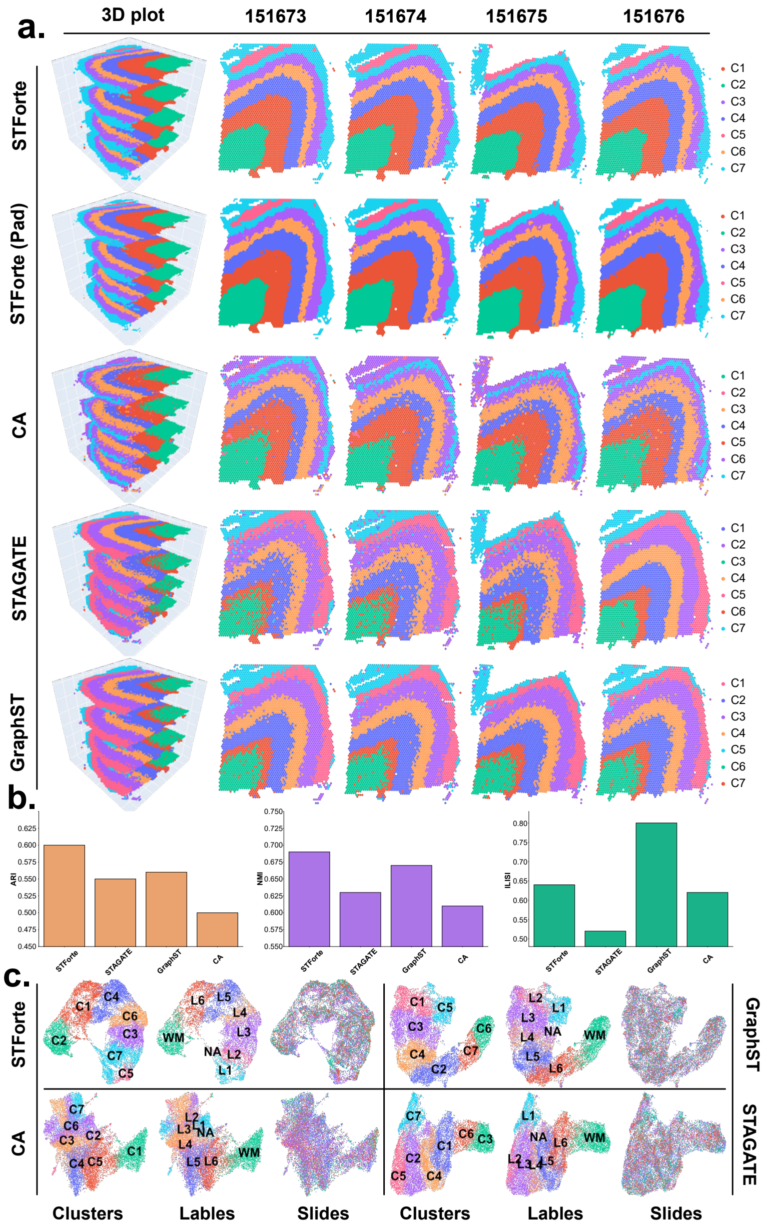

Supplementary Fig. S15 a, Spatial region identification results for the DLPFC dataset (No. 151673-151676) using different methods in multi-slice scenarios. **b,** Comparative performance of different methods in multi-slice analysis, including averaged ARI, averaged NMI, and iLISI. **c,** UMAP visualizations of multi-slice data obtained by different methods.

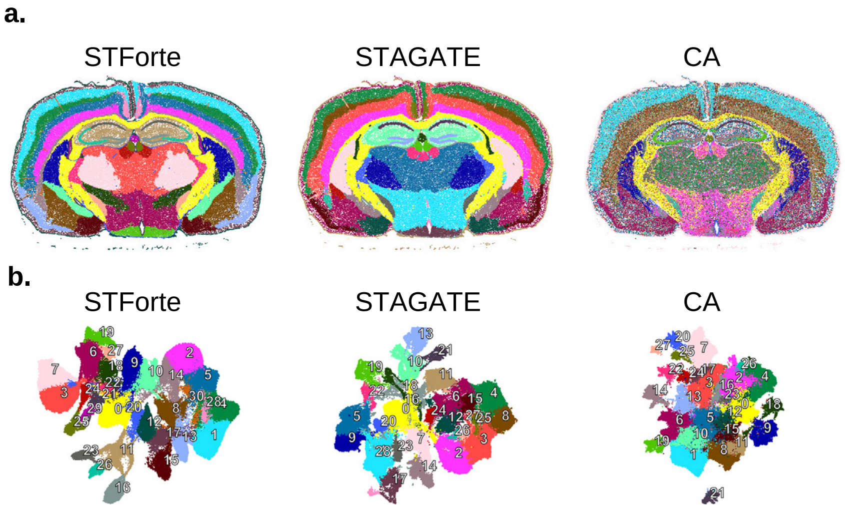

Supplementary Fig. S16 a, Spatial regions and **b,** UMAP visualization identified by different methods for 10x Xenium mouse coronal brain dataset.

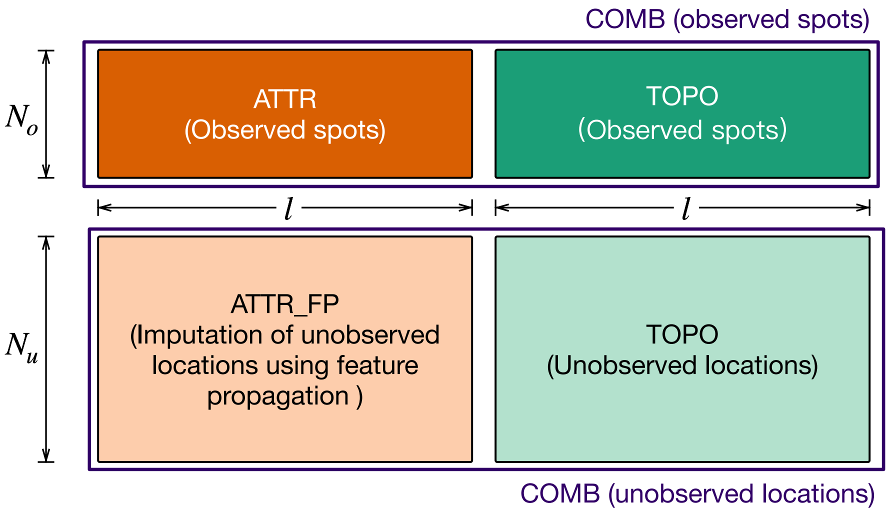

Supplementary Fig. S17 Schematic description of the encodings in the STForte model. The trained model generates attribute encoding (ATTR) and topology encoding (TOPO). ATTR contains only the patterns of observed spots, whereas TOPO additionally includes imputed information for unobserved locations. Furthermore, Feature propagation is performed to impute the ATTR encoding of unobserved locations. By concatenating ATTR and TOPO encodings, we obtain combined encodings (COMB), which provide more homogenized spatial information for downstream analysis compared to ATTR. TOPO demonstrates superior consistency between observed and unobserved locations, which facilitates its application for the property propagation of unobserved encodings. N_o: data size of observed spots; N_u: data size of unobserved locations; l: dimension of each latent encoding.

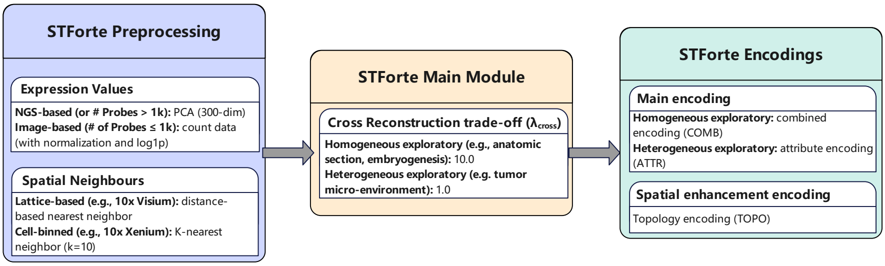

Supplementary Fig. S18 Recommended recipe of STForte for user guidance. During the preprocessing stage, users can decide whether to perform PCA based on the number of probes in the spatial omics data (*e.g.*, number of genes) and choose between distance-based nearest neighbor (NN) and KNN according to the spatial morphology of the data. When training the model, users can select the cross-reconstruction parameter based on the task on which they wish to focus, such as homogeneity (*e.g.*, anatomical partitioning) or heterogeneity (*e.g.*, tumor micro-environment). When selecting the main encoding, attribute (ATTR) or combined (COMB) encodings can be chosen according to the types of exploratory tasks. Topology (TOPO) encoding is recommended for spatial enhancement.

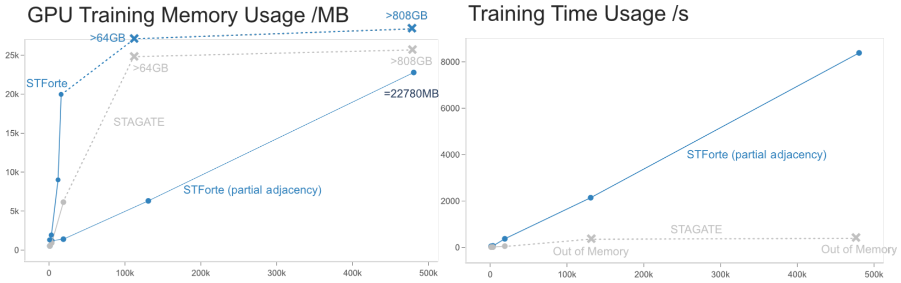

Supplementary Fig. S19 Computational efficiency compassion. Without any computational optimization, the memory consumption of the base STForte model quickly reaches the maximum capacity (24GB) of the single GPU we used. Additionally, the rate of GPU memory increase is higher than that of methods like STAGATE. The partial adjacency trick can reduce this memory consumption significantly. This optimization enables STForte to handle large single-cell resolution datasets with 100,000 to 500,000 cells/spots on a single 24GB GPU.

Supplementary Tables

	ONL	GL	EPL	MCL	GCL	Excluded
**Fabp7 (ONL)**	1.00E + 00	6.48E − 48	6.32E − 60	2.04E − 44	9.62E − 65	2.75E − 98
**Nrsn1 (GL)**	3.47E − 41	1.00E + 00	2.71E − 39	1.43E − 28	1.89E − 52	1.74E − 71
**Bc1 (EPL)**	2.84E − 45	2.06E − 52	1.00E + 00	1.17E − 33	7.86E − 52	4.52E − 70
**Lbhd2 (MCL)**	7.16E − 23	3.12E − 22	2.85E − 09	1.00E + 00	8.01E − 24	6.36E − 25
**Meis2 (GCL)**	8.51E − 67	2.18E − 64	1.66E − 53	5.82E − 35	1.00E + 00	1.66E − 86

Supplementary Table S1 One-sided Wilcoxon rank-sum test p-values of 10x Visium mouse olfactory bulb data. Rank-sum tests are conducted for the layer-specific marker gene of corresponding layer in interest compared with other clusters or the entire set excluded the layer in interest. The results show significant divergence for the expression level of marker genes (p-values $<$ 1E-20).

	ONL(5)	GL(3)	EPL(0)	MCL/IPL(4)	GCL1(1)	GCL2(2)	RMS(6)
**Fabp7**	8.59E − 166	6.64E − 24	ns	ns	ns	ns	ns
**Nrsn1**	ns	1.47E − 34	8.68E − 12	ns	ns	ns	ns
**Bc1**	ns	ns	6.54E − 05	5.73E − 04	ns	ns	ns
**Lbhd2**	ns	ns	ns	8.87E − 08	ns	ns	ns
**Pcp4**	ns	ns	ns	1.73E − 13	9.48E − 280	4.46E − 110	ns
**Gad1**	ns	ns	ns	4.57E − 41	4.36E − 172	1.63E − 13	ns
**Meis2**	ns	ns	ns	ns	3.54E − 90	2.42E − 63	2.07E − 15
**Mbp**	ns	ns	ns	ns	7.76E − 11	2.35E − 08	1.42E − 37

Supplementary Table S2 One-sided Wilcoxon rank-sum test p-values of Stereo-seq mouse olfactory bulb data. Rank-sum tests are conducted for the layer-specific marker gene under a specific layer compared with other layers (i.e., the entire set excluded the layer in interest). P-value > 1E-3 will be considered as non-significant (ns).

	ARI			NMI
	signed-rank	ranksum	signed-rank	ranksum
**COMB-ATTR**	0.6221	0.9540	0.4697	0.8174
**COMB-TOPO**	0.0005	0.007	0.0005	0.0001
**ATTR-TOPO**	0.0049	0.0039	0.0005	0.0003

Supplementary Table S3 P-values of Wilcoxon signed-rank tests and Wilcoxon rank-sum tests for the clustering performance metrics based on different STForte encodings. The investigation was conducted across 12 slices in the 10x Visium DLPFC dataset.

**Method**	**ARI**		**NMI**	
	signed-rank	ranksum	signed-rank	ranksum
**STAGATE**	0.4697	0.4189	0.0522	0.1482
**GraphST**	0.5186	0.6861	0.0522	0.3263
**BANKSY**	0.8501	0.4884	0.6772	0.6442
**BayesSpace**	0.0010	0.0647	0.0161	0.2040
**DeenST**	0.0427	0.0833	0.3013	0.6422
**SpaGCN**	0.0923	0.0240	0.0093	0.0833
**CA**	0.0342	0.0243	0.0010	0.0003
**spaVAE**	0.0522	0.0377	0.0049	0.0067
**scVI**	0.0005	0.0008	0.0005	0.0001
**scanpy**	0.0005	0.0001	0.0005	$< 1e-6$

Supplementary Table S4 P-values of Wilcoxon signed-rank tests and Wilcoxon rank-sum tests for the clustering performance metrics based on other methods versus STForte. The investigation was conducted across 12 slices in the 10x Visium DLPFC dataset.

Cluster	Domain	Name
1	Cerebral cortex	L2/3
2	Cerebral cortex	L6
4	Cerebral cortex	L4
5	Cerebral cortex	L5
8	Cerebral cortex	Cortical subplate
13	Cerebral cortex	Piriform
14	Cerebral cortex	L6b
15	Cerebral cortex	Cortical amygdalar area (CAA)
17	Cerebral cortex	L1
28	Cerebral cortex	
30	Cerebral cortex	
9	Cerebral nuclei	Striatum dorsal region
10	Cerebral nuclei	Striatum-like amygdalar nuclei
0	Fiber tracts	Fiber tracts
11	Hippocampal	Hippocampal stratum
16	Hippocampal	DG-sg (Dentate gyrus, granule cell layer)
23	Hippocampal	CA1sp (Field CA1, pyramidal layer)
26	Hippocampal	CA2sp/CA3sp (Field CA2/3, pyramidal layer)
6	Hypothalamus	
18	Hypothalamus	Zona incerta
19	Hypothalamus	
27	Hypothalamus	[Subthalamic nucleus]
12	Meninges	
20	Meninges	
21	Meninges	
3	Thalamus	Polymodal association cortex related
7	Thalamus	Sensory-motor cortex related
22	Thalamus	Reticular nucleus of the thalamus
24	Thalamus	Epithalamus (Lateral habenula)
25	Thalamus	Epithalamus (Medial habenula)
29	Ventricular	

Supplementary Table S5 Region annotations of 10x Xenium mouse coronal brain dataset according to the spatial and anatomical characteristics derived from STForte spatial identification results.

	L1	L2/3	L4	L5	L6	L6b	C28
**Nrep**	1.00	$< 1e-6$	$< 1e-6$	$< 1e-6$	1.00	1.00	$< 1e-6$
**Ccn2**	1.00	1.00	1.00	0.24	$< 1e-6$	$< 1e-6$	1.00
**Rprm**	1.00	1.00	1.00	$< 1e-6$	$< 1e-6$	0.96	1.00
**Fezf2**	1.00	1.00	1.00	$< 1e-6$	$< 1e-6$	$< 1e-6$	0.06
**Rorb**	$< 1e-6$	1.00	$< 1e-6$	1.00	1.00	1.00	1.00
**Rasgrf2**	1.00	$< 1e-6$	$< 1e-6$	1.00	1.00	1.00	1.00
**Gfap**	$< 1e-6$	1.00	1.00	1.00	$< 1e-6$	$< 1e-6$	1.00

Supplementary Table S6 One-sided Wilcoxon rank-sum test p-values of isocortex domain within 10x Xenium mouse coronal brain dataset. Rank-sum tests are conducted for the layer-specific marker gene under a specific layer compared with other layers (i.e., the entire set excluded the layer in interest).

	**Hipp. Stratum**	**CA1sp**	**CA2sp/CA3sp**	**Dg-sg**
**Prox1**	1.000	1.000	1.000	$< 1e-6$
**Neurod6**	1.000	$< 1e-6$	$< 1e-6$	1.000
**Wfs1**	0.002	$< 1e-6$	1.000	1.000
**Cpne4**	1.000	1.000	$< 1e-6$	$< 1e-6$

Supplementary Table S7 One-sided Wilcoxon rank-sum test p-values of hippocampal domain within 10x Xenium mouse coronal brain dataset. Rank-sum tests are conducted for the marker gene under a specific region compared with other layers (i.e., the entire set excluded the region in interest).

Parameter	Default	10x Visium MoB	10x Visium DLPFC	10x Visium OSCC	10x Visium Prostate Cancer	Stereo-seq MoB	10x Xenium Mouse Brain
STForte Preprocessing							
Spatial neighbor	Depends on spatial formality	distanced-nearest neighbor (NN)				KNN (k = 18)	KNN (k = 10)
PCA for expression values	300-dim	-	-	-	-	-	Raw input
STForte Main Module							
Hidden layer size	[200, 50]	-	-	[100, 50]	-	-	-
Cross reconstruction weight (λ_{cross})	10.0	-	-	1.0	1.0	-	-
Adversarial distribution matching weight (λ_{adv})	4.0	-	-	-	-	-	-
Training epochs	450	-	-	-	-	500	500
STForte Encodings							
Main encoding	COMB	-	-	ATTR	ATTR	-	-
Spatial enhancement encoding	TOPO	-	COMB	-	-	-	-

Supplementary Table S8 Parameter selection for STForte across different implementations. A dash ("-") indicates where the default parameter (as in **Default**) was used in a particular experiment.

dataset	scale	model	pre-time(r)/s	pre-time/s	pre-mem/Mb	train-time/s	train-gpu-mem/Mb
OSCC	1131	STForte	2.69	5.02	922.99	46.79	526
OSCC(PAD)	4096	STForte(PAD)	4.88	7.97	922.99	78.25	1150
DLPFC151673	3639	STForte	6.12	21.83	2130.83	62.38	1196
DLPFC151673(PAD)	14201	STForte(PAD)	21.23	41.93	2130.83	185.98	8594
Stereo-seq Olfa(KDT)	19109	STForte	10.64	12.1	2320.29	386.73	20268
Stereo-seq Olfa	19109	STForte	29.66	30.73	2533.94	386.11	20268
Stereo-seq Olfa(KDT-partialADJ)	19109	STForte	11.03	12.32	2320.36	369.88	1392
Xenium Brain (partialADJ)	130870	STForte	17.89	nan	1138.86	2140.41	6306
Vizgen Liver Cancer (partialADJ)	480592	STForte	78	nan	5601.5	8383.09	22780
OSCC	1131	STAGATE	nan	2.54	112.33	10.98	516
DLPFC151673	3639	STAGATE	nan	3.01	306.43	12.82	914
Stereo-seq Olfa	19109	STAGATE	nan	13.3	12710.39	53.43	6144
Xenium Brain	130870	STAGATE	nan	71.78	851.94	nan	>65536
Vizgen Liver Cancer	480592	STAGATE	nan	260.53	3274.72	nan	>881080.32

Supplementary Table S9 Computational efficiency of STForte compare to STGATE. Nan in the preprocessing time columns indicates >500s or the metric is not applicable. KDT means using KD-tree to accelerate the KNN graph construction. Nan in the GPU training time column means out of memory.

Supplementary Notes

1 Graph Construction and Padding Strategy

1.1 Neighborhood Graph

STForte offers two graph construction approaches according to the context of different ST technologies. For technologies that generate regular or approximately regular spot lattices, such as Visium or Ståhl *et al.*, neighborhood graphs are preferred in their original resolution. In this case, the distance parameter d corresponds to the distance between adjacent spot centers in the regular lattices.

In situations where the distance parameter is not provided or the data exhibits approximately regular spot lattices, STForte can estimate the spot distance by utilizing an expected number of neighbors n and an error bound ϵ. The estimation process involves randomly sampling a spot from the dataset and assessing the differences in distances between the nearest n spots and the sampled spot. If all of these differences fall within the specified ϵ, the maximum value of these n distances is recorded. Conversely, if any of the differences exceed ϵ, a failure trial is noted. This iterative process continues until a predetermined number of iterations is reached. If the majority of trials yield bounded differences, the highest recorded distance is considered the estimated spot distance.

With a defined or inferred value for d, the neighborhood graph is subsequently defined, wherein the neighbor set of a given spot i consists of all spots located within a Euclidean circle of radius d centered at the location of spot i, which is:

$$\mathcal{N}(i) = \{j; d(i,j) \leq d \; \forall j = 1, \ldots, N\}, \tag{1}$$

where $d(i,j)$ is the Euclidean distance between the locations of spot i and j.

1.2 KNN Graph

STForte applied KNN graphs for ST data with irregular lattice, which is a commonly encountered scenario in single-cell resolution datasets. In order to construct a KNN graph, a fixed value for K which represents the number of neighbors must be specified. For each individual cell, Euclidean distance is measured between its location and the locations of all other cells within the dataset, and the K closest cells are selected to form the neighborhood set of that particular cell. Mathematically speaking, the neighbor set of cell i is defined as

$$\mathcal{N}(i) = \{j; d_{(k)}(i,j) \; \forall j = 1, \ldots, N \; and \; k \leq K\}, \tag{2}$$

where the $d_{(k)}(i,j)$ denotes the kth ordered distance.

1.3 Padding Strategy

The capability of STForte to infer the expression profile of unobserved locations has inspired the exploration of super-resolution using the platform through manual generation of pseudo spots. The concept is based on the observation that in Visium, there is a $45\mu m$ gap between spots, approximately equivalent to the diameter of a spot. Thus, the insertion of pseudo spots between each neighboring spot pair represents a potential method for achieving higher resolution. This approach involves the insertions of pseudo spots at the midpoint of each edge of the neighborhood graph constructed following the process outlined in the Section 1.1 and then infer the expression profiles of those pseudo spots using STForte.

2 Pathfinder discovery network

In order to encode the topology information, STForte utilizes a Pathfinder Discovery Network (PDN) [1] for the topology encoder. Specifically, denote $\mathbf{H} \in \mathbb{R}^{n\times d_{\text{in}}}$ as the input and $\mathbf{H}' \in \mathbb{R}^{n\times d_{\text{out}}}$ as the output of a PDN layer and $\tilde{\mathbf{A}} \in \mathbb{R}^{n\times n}$ is the weighted adjacency matrix, where n, d_{in} and d_{out} are the node size (i.e, the number of nodes), input dimension and output dimension, respectively. The message passing operation can be calculated as follows:

$$\mathbf{H}' = \hat{\sigma}(\mathbf{D}_{\hat{\mathbf{G}}}^{-\frac{1}{2}}\hat{\mathbf{G}}\mathbf{D}_{\hat{\mathbf{G}}}^{-\frac{1}{2}}\mathbf{H}\mathbf{W} + \mathbf{b}) \tag{3}$$

$$\hat{\mathbf{G}}_i = g_\Omega(\tilde{\mathbf{A}}_i) \tag{4}$$

where g_Ω is an multi-layer perceptron (MLP) operator. $\mathbf{D}_{\hat{\mathbf{G}}}$ is the diagonal degree matrix of $\hat{\mathbf{G}}$. $\mathbf{W}$ is a learnable weight matrix and $\mathbf{b}$ is the bias vector. $\hat{\sigma}$ is an activation function.

While STForte accommodates the input of spatial distances among spots (cells) as a weighted adjacency matrix, it is important to note that in its default configuration, only the neighbor information between spots (cells) are utilized as input for the adjacency matrix (i.e., $\mathbf{A} \in \{0,1\}^{n\times n}$). Under these circumstances, the PDN is functionally equivalent to a Graph Convolutional Network (GCN) [2], with calculation as follows:

$$\mathbf{H}' = \hat{\sigma}(\mathbf{D}^{-\frac{1}{2}}\hat{\mathbf{A}}\mathbf{D}^{-\frac{1}{2}}\mathbf{H}\mathbf{W} + \mathbf{b}) \tag{5}$$

where $\hat{\mathbf{A}} = \mathbf{A} + \mathbf{I}$ is the adjacency matrix with inserted self-loops. $\mathbf{D}$ is the diagonal degree matrix.

Moreover, to introduce nonlinearity and prevent overfitting, the rectified linear unit (ReLU) [3] activation function and dropout regularization [4] technique are incorporated into the graph neural network architecture.

3 Feature propagation

STForte also adopts a feature propagation (FP) [5] method to impute the unseen expression patterns from unobserved locations. FP intends to minimize the Dirichlet energy to achieve diffusion-based feature reconstruction through an iterative optimization. Specifically, $\mathbf{x} \in \mathbb{R}^N$ is a feature vector and $\mathbf{A} \in \{0,1\}^{N\times N}$ for a graph with N denotes the number of nodes in total. Following with the statements of STForte, we can assume that the $\mathbf{x}$ is composed by $\mathbf{x}_o \in \mathbb{R}^{N_o}$ for observed spots and $\mathbf{x}_u \in \mathbb{R}^{N_u}$ for unobserved spots. Therefore, the ordering of nodes are arranged as follows:

$$\mathbf{x} = \begin{bmatrix} \mathbf{x}_o \\ \mathbf{x}_u \end{bmatrix}, \mathbf{A} = \begin{bmatrix} \mathbf{A}_{oo} & \mathbf{A}_{ou} \\ \mathbf{A}_{uo} & \mathbf{A}_{uu} \end{bmatrix} \tag{6}$$

where $\mathbf{A}$ is as a partitioned matrix with different blocks are considered as the sub-adjacent matrix for observed-observed nodes ($\mathbf{A}_{oo}$), observed-unobserved nodes ($\mathbf{A}_{ou}$ and $\mathbf{A}_{uo}$ with $\mathbf{A}_{ou}^\top = \mathbf{A}_{uo}$) and unobserved-unobserved nodes ($\mathbf{A}_{uu}$), respectively. To this end, denote the normalized adjacent matrix as $\mathbf{A}' = \mathbf{D}^{-\frac{1}{2}}\mathbf{A}\mathbf{D}^{-\frac{1}{2}}$ with $\mathbf{D}$ being the degree matrix of $\mathbf{A}$, we can iteratively update $\mathbf{x}$ as follows:

$$\mathbf{x}^{(k+1)} = \begin{bmatrix} \mathbf{I} & 0 \\ \mathbf{A}'_{uo} & \mathbf{A}'_{uu} \end{bmatrix} \mathbf{x}^{(k)} \tag{7}$$

where $\mathbf{A}'_{uo}$ is the sub-adjacent matrix for the connection from unobserved spots to observed spots and $\mathbf{A}'_{uu}$ represents the sub-adjacent matrix from the unobserved spots.

The FP method was implemented in two distinct parts throughout our experiments:

(i) Within the main infrastructure of STForte, FP was employed to perform initial interpolation of expression profiles at unobserved locations to learn the topology encoding.

(ii) In our comparative analysis, FP was applied to attribute encoding to generate corresponding interpolations for unobserved spots, thereby enabling evaluation and visualization of the representational capacity and imputation capabilities of different encodings.

4 Encodings and property propagation

The trained model generates two encodings from the corresponding SRT data: attribute encoding (ATTR, Z_X) to encode the expression pattern information in the observed spots, and topology encoding (TOPO, Z_A) to compress the spatial patterns for both the observed spots and spots with unobserved transcripts. For unobserved locations, feature propagation is adopted to impute the ATTR encoding. In addition, ATTR and TOPO encodings are further concatenated to formulate combined encodings (COMB).

The generated encodings can be utilized for various downstream analyses of the SRT data. COMB encodings can be more effective for spatial region identification or related analyses of consecutive tissue domains (e.g., anatomical regions) by considering a trade-off between expression patterns and spatial connectivity. For the analyses of tissues with higher complexity and heterogeneity, ATTR encoding is more suitable for identifying distinct patterns by mitigating the influence of spatial connectivity. In addition, the trade-off of spatial connectivity can also be handled implicitly through adjusting the cross-reconstruction factor λ_{cross}.

To enable fine-grained analysis of SRT data, STForte includes additional utilities for propagating annotation or imputing gene expression levels for unobserved spots. Specifically, the latent encoding of the observed spots serves as training data to fit the XGBoost model [6]. Subsequently, the properties of unobserved spots are predicted using the fitted XGBoost. Annotation propagation is handled as a classification task, whereas gene imputation is a regression task used to predict the expression levels of specific genes. Normalization of gene expression levels included log-transformation and normalization according to library size from the Scanpy package [7]. Because TOPO encoding considers more consecutive information about spatial connectivity, it is adopted as encoding for propagation or imputation by default in analyses. Note that other encodings are also included in the investigation and evaluation of the encodings in the result sections.

5 Details on analysis

We ensured utmost uniformity in the preprocessing and the selection of hyperparameters across different datasets. However, slight variations may be present owing to differences in the SRT techniques and spatial heterogeneity. Here, we describe the details of analysis according to different investigated datasets as follows.

5.1 10x Visium mouse olfactory bulb dataset

Lebrigand et al. [8] processed olfactory bulb tissue samples from C57BL/6 mice (>2 months old) using the 10x Genomics Visium protocol. The original dataset comprised 918 spots and 31,053 genes. Spatial neighbors were constructed based on the distance-based strategy, which is the default setting for 10x Visium datasets in STForte (Supplementary Note S1). Outlier cells expressing < 200 genes were masked as unobserved locations. Additionally, padding strategy was used to identify gaps within the original spots as unobserved locations. PCA with 300-dimensions was applied for the pre-dimensional reduction of the expression counts using "sc.pp.pca" from the Scanpy library. Subsequently, STForte was applied to the preprocessed data using default hyperparameters. For spatial region identification, "mclust", with the number of clusters set to six, was applied to different STForte encodings. The primary investigations and visualizations were performed using COMB encoding for the observed spots, taking into account the spatial continuity of the laminar structure in the olfactory bulb.

5.2 Stereo-seq mouse olfactory bulb dataset

The adult mouse olfactory bulb data obtained by Stereo-seq was binned to a resolution approximating the cellular level ($\approx$14 μm) [9]. We retained only on-tissue cells based on the corresponding cell quality indices [10]. Subsequently, cells with defective expression profiles were masked (i.e., identified as unobserved locations) according to the number of expressed genes for each cell and the provided cell quality indices. The resulting dataset contains 19,326 cells (including unobserved locations) and 22,780 genes. Considering that the number of cells exceeded 15k, PCA based on the PyTorch [11] backend was used to generate a 300-dimensional pre-dimensional reduction for the expression profiles. In addition, a k-nearest neighbor (KNN)-based strategy ($k = 18$) was employed to construct spatial neighbors, taking into account the non-lattice identity of the dataset. STForte was applied to the preprocessed data using default hyperparameters, except for the sparse manner in the reconstruction of the adjacency matrix, considering the size of the dataset. Consequently, Leiden clustering [12] ("sc.tl.leiden" from Scanpy) with a resolution of 0.3 was adopted for spatial region identification. The primary investigations and visualizations were proceeded with COMB encoding.

5.3 Prostate adenocarcinoma dataset

The 10x Visium spatial formalin-fixed, paraffin-embedded (FFPE) sample of prostate adenocarcinoma was obtained from the 10x Genomics repository, which contains 4,371 spots and 17,943 genes. The preprocessing steps for the dataset were the same as those used for the 10x Visium mouse olfactory bulb data, with the only difference being the exclusion of outlier spot filtering due to the relatively intact expression profile quality across this sample. STForte was applied with $\lambda_{\text{cross}} = 1$ (default setting is 10) considering the heterogeneity of the tumor micro-environment (TME). Leiden clustering was employed for spatial region identification. We investigated the clustering results by varying the resolution from 0.1 to 1 with a step size of 0.05 and selected the best results based on the highest silhouette coefficient. Primary investigations and visualizations were performed using ATTR encoding, considering the heterogeneity of TME. Furthermore, we applied COMMOT [13] to investigate spatial cell-cell communications based on the STForte results.

5.4 Human OSCC dataset

The OSCC dataset contained 12 frozen and optimal cutting temperature embedded tissue samples and spatial transcriptomics was performed using 10x Visium [14]. Each data sample contains 1200 spots and 16,000 genes. Genes that were not detected in any spot were removed during the preprocessing and then followed by dimensional reduction to 300 via PCA. Spatial enhancement preparation and graph construction were accomplished using KNN reconstruction with hyperparameter k=18. Then STForte was applied to the prepared data with the neurons in the two GNN layers reduced to 100 and 50 respectively, and $\lambda_{cross} = 1$. Spatial domain identification was achieved through the Louvain algorithm based on the attribute encodings.

5.5 Human DLPFC dataset

The famous six-layered human dorsolateral prefrontal cortex (DLPFC) dataset included 3 groups of samples (4 continuous sections per group) generated using the 10x Visium technique [15]. The pre-processing, dimensional reduction and graph construction schemes of the DLPFC dataset were the same as those dealing with the OSCC dataset. Two larger GNN layers of 200 and 50 neurons and $\lambda_{cross} = 10$ were adapted during the training process. Spatial domain identification was performed over concatenated attribute and topology encodings using R package 'mclust'. Cluster numbers were explicitly set to 7 for samples in group 1 (151507-151510) and group 3 (151673-151676), and 5 for samples in group 2 (151669-151672). For multi-slices analysis, PASTE-v2 [16] was first applied to align the 2D coordinates of different samples in one group, followed by the same pre-processing and dimensional reduction scheme. The cross-slices distance-based neighbor graph was constructed with d=145, then the STForte model with the same parameters as training on a single slice. Clustering adapted "mclust" package and spatial enhancement were done by

extending the multi-slices clustering results over the topological encodings of each single-slice model.

5.6 10x Xenium mouse brain dataset

10x Genomics obtained a 10μm fresh frozen tissue section of the coronal brain from a C57BL/6 mouse, which was prepared following the 10x Xenium In Situ protocol [17]. The dataset is publicly available in the 10x Genomics repository and contains 130,870 cells and 248 genes. During preprocessing, cells with counts less than 10 were excluded using "sc.pp.filter_cells" from the Scanpy library. Expression counts were normalized according to their total counts across all genes and log-transformed. The processed expression profiles were used as direct inputs for STForte. Spatial neighbors were constructed using the KNN-based strategy with $k = 10$. STForte was applied to the preprocessed data in a sparse manner, with 500 epochs for the training process. Subsequently, Leiden clustering with a resolution of 0.85 was employed for spatial region identification. Investigations and visualizations were performed using the COMB encoding.

6 Evaluation Metrics

This section discusses the formula and implementation of the metrics used in this study.

6.1 Clustering metrics

This study utilized four metrics to quantify the clustering performance including Adjusted Rand Index (ARI), Normalized Mutual Information (NMI), Silhouette Coefficient (SC), Calinski-Harabaz Score (C-H), and Local Inver Simpson's Index (LISI). The first two metrics were used to demonstrate the clustering performance when the true cluster labels were known. The following two metrics were considered to quantify the performance of different methods in real exploratory data analysis. The last metric LISI was used to quantify the performance of multi-slices clustering results. The first four metrics were calculated using the function in the metric module of package Sci-kit Learn and the LISI was implemented via package scib through the interface provided by Scanpy.

6.1.1 Adjusted Rand Index (ARI)

ARI is a statistical measure that quantifies the similarities between two clustering labels of the same dataset. ARI picks a value between -1 and 1, where 1 indicates perfect agreement and -1 implies the counterproductive result. The calculation of ARI extends the Rand Index (RI) by adjusting for the expected agreement that would occur by chance, which could be formalized as

$$ARI = \frac{RI - E[RI]}{Max(RI) - E[RI]} \tag{8}$$

where RI computes the similarities between two clustering labels by considering all pairs of samples and counting disagreement pairs, which is

$$RI = \frac{N_{agree}}{N_{total}} \tag{9}$$

Where N_{agree} denotes the number of agreed assignments and N_{total} indicates the total number of pairs between two clusters.

6.1.2 Normalized Mutual Information (NMI)

NMI is another statistical measurement that quantifies the same goal as ARI. It serves as a normalized version of mutual information score (MI) by dividing the generalized mean of entropies of the two cluster assignments, which is defined as follows,

$$NMI(U, V) = \frac{2MI(U, V)}{H(U) + H(V)} \tag{10}$$

where U and V denote the two cluster assignments. $H(\dot{})$ calculates the entropy, and $MI(U, V)$ represents the mutual information between cluster assignments. As a result, NMI ranges from 0 to 1, where a higher value indicates a better agreement between two cluster assignments.

6.1.3 Silhouette Coefficient (SC)

SC [18] measures the separation among clusters which takes a value from -1 to 1. A higher value of SC indicates the clusters are more separated from each other which implies a more reasonable clustering result. Given the clustering assignment, SC first computes the intra-cluster distance denoted by d_{itra} and the minimum of inter-cluster distance denoted by d_{inter}. Then SIL is defined as

$$SC = \frac{d_{inter} - d_{intra}}{max(d_{inter}, d_{intra})} \tag{11}$$

6.1.4 Calinski-Harabaz Score (C-H)

The C-H, also known as the Variance Ratio Criterion, is a statistical measure of the separation of clusters [19]. It is particularly useful when comparing clustering performance when the number of clusters of clustering labels is different. Essentially, the C-H compares the ratio of the distance between the cluster centers to the average distance of points within a cluster. The formula of the C-H is

$$C - H = \frac{tr(S_b)}{tr(S_w)} \times \frac{n-k}{k-1} \tag{12}$$

where S_b is the between-cluster scatter matrix, S_w is the within-cluster scatter matrix, n is the total number of samples, and k is the number of clusters. A higher value of C-H indicates that the clusters are well-defined and well-separated from each other.

6.1.5 Integration Local Inverse Simpson's Index (iLISI)

Local Inverse Simpson's Index (LISI) [20] is an extension of Simpson's Index (SI) which could be calculated as

$$LISI = \frac{1}{\sum_{i=0}^{K} p_i^2} \tag{13}$$

where p_i is the proportion that the cluster label i is in the local neighborhood, and K is the total number of some categorical effects. LISI accesses whether cells are well-mixed across some categorical variables, like batch, technologies, and donor. Traditional LISI takes a value from 0 to number of batches and the iLISI used in this study scales the result to a number from 0 to 1 but does no affect the trend of LISI. This study utilized iLISI to compare the batch removal performances of different methods. A higher value of iLISI indicates a better mixture of batches which means better batch removal results.

6.2 Other Metrics

STForte as a representation learning model with spatial enhancement ability, this study used multiple metrics to quantify its downstream analysis abilities, including Mean Squared Error (MSE), and Accuracy Score (ACC). This study used MSE to numerically demonstrate the performance of recovering gene expression of unobserved spots using different methods. ACC was calculated to assess whether these methods could precisely recognize the spots that should have non-zero expression value by recovering their expression level ≥ 0.5 These two metrics were calculated through the metrics module of Scikit-Learn.

7 Compared Methods

7.1 STAGATE

STAGATE [10] is a computational model designed for spatial transcriptomics data analysis, integrating gene expression with spatial information through a graph attention auto-encoder (GAE) framework to identify spatial domains and denoise data. Different from the traditional GAE framework, STAGATE replaces the outer-product decoder by a transposed GNN module whose parameters are copied from the encoder to reconstruct the gene expression. Experimentally, this study followed the official tutorial (https://stagate.readthedocs.io/en/latest/) to train STAGATE over 10xVisium and multi-slices 10x Visium data. The results demonstrated using Stereo-seq and Xenium data imitated the procedure for analyzing Slide-seq data with refined radius cutoff to ensure each spot gets approximately 10 neighborhoods on average.

7.2 GraphST

GraphST [21] proposed a self-supervised graph contrastive learning framework for exploiting spatial information together with gene expression profiles. It is one of the current benchmarks of spatial domain identification. The clustering results of 10x Visium data produced in this study follow the procedures presented in the official tutorial of GraphST (https://deepst-tutorials.readthedocs.io/en/latest/). The multi-slide results were done by applying Paste2 to align the spatial locations first before getting into the official procedure.

7.3 BayesSpace

BayesSpace [22] is a fully Bayesian statistical approach that ingeniously utilizes neighborhood structures in spatial transcriptomic data, allowing for accurate clustering, differential expression analysis, and enhancement of spatial resolution. In this study, the DLPFC clustering result was produced exactly following the same settings in its original tutorial (http://www.ezstatconsulting.com/BayesSpace/articles/maynard_DLPFC.html). The spatial resolution enhancement results were calculated using the same procedure and parameter settings in the demonstration of SCC (http://www.ezstatconsulting.com/BayesSpace/articles/ji_SCC.html).

7.4 DeepST

DeepST [23] is also a graph-based representation learning method that incorporates the GAE framework with an additional expression decoder. DeepST also provides a more fine-grained procedure for expression preprocessing and constructing the spatial graph. The clustering result of the DLPFC of DeepST was constructed under the instructions shown on its GitHub pages (https:

//github.com/JiangBioLab/DeepST) with slight modifications due to the different versions of package dependencies.

7.5 SpaGCN

SpaGCN [24] integrates spatial information, gene expression, and histological image via graph convolutions and produces comprehensive embeddings for SRT data. Like STForte, SpaGCN uses the top PCA components as model input instead of dealing with raw counts directly. In the comparison part of this study, the result of SpaGCN was produced following its tutorial (https://github.com/jianhuupenn/SpaGCN/blob/master/tutorial/tutorial.md) but without the consideration of histological image for fairness (All other methods do not need histological information for embeddings).

7.6 CA

The Correspondence Analysis (CA) [25] is not a popular method in traditional single-cell and SRT data analysis. However, due to our analysis experience, this method is simple but usually efficient and effective. The CA is a matrix factorization method that serves as an alternative to PCA on count data. Different from PCA which approximates the Euclidean distance for differences in embeddings, CA performs the decomposition over the overall chi-squared statistic matrix. As a result, CA could theoretically avoid the unsatisfied continuous Gaussian assumption of PCA. The original article of CA provides source codes in R and this study translated the canonical form CA into Python codes and embedded it in the STGraph module.

7.7 scVI

scVI [26] is a deep-learning-based non-spatial embedding method that is popular for dealing with single-cell data. It adopts a variational auto-encoder framework with considerations of library size and batch effects. This study followed the guidelines of the package scvi-tools (https://docs.scvi-tools.org/en/stable/tutorials/index.html) to generate the clustering result by treating the SRT data as traditional single-cell data.

7.8 BANKSY

BANKSY [27] is a spatial kernel based method that utilize two different spatial kernels to enhance the gene expression profile of each spot/cell to achieve more representative spatial domain identification results.We strictly followed the guidance in the manuscript and supplementary materials for benchmarking on BANKSY (https://github.com/prabhakarlab/Banksy). The gene expression vector of each spot was normalized by the median of all library sizes across spots, and the top 2000 genes were selected for downstream modeling using the traditional Seurat method. Then, the truncated gene expression

matrix was renormalized again with the new median of library size and transformed using log1p. Both the averaged neighbor kernel and the AGF kernel were used for space lifting. Then, all three matrices were concatenated with a scaling factor =0.2 as suggested by the manuscript. After performing PCA to reduce the concatenated matrix to dimensions of 20, the Leiden algorithm was applied for clustering with resolution increased from 0.1 to 1.5 and step 0.02. The ARI and NMI were calculated as the median values of all clustering results with the same number of clusters as the true labels.

7.9 spaVAE

spaVAE [28] uses a deep learning based variational inference structure with negative binomial distribution as inference family to perform spatial domain identification as well as spatial resolution enhancement. To benchemark spaVAE, the training with the maximum iteration of 5000 took about 2 hours on a single slide of DLPFC data using our computer. This makes spaVAE incompatible with other benchmark methods, which usually take a few minutes for training on datasets of this scale, despite spaVAE reported that they achieve comparable performance. Therefore, by taking a closer look at the loss track of the training process, we found the model only got marginal improvement in its total training loss after iteration 500. To achieve the performance-resource balance, we decided to change the maximum iterations to 500 and preserve all the other hyperparameters suggested by the GitHub tutorial (https://github.com/ttgump/spaVAE) provided by their paper. Therefore, the presented performance of spaVAE may not be their best performance but still meaningful as a reference.

References

[1] Benedek Rozemberczki, Peter Englert, Amol Kapoor, Martin Blais, and Bryan Perozzi. Pathfinder discovery networks for neural message passing. In *Proceedings of the Web Conference 2021*, WWW '21, page 2547–2558, New York, NY, USA, 2021. Association for Computing Machinery.

[2] Thomas N. Kipf and Max Welling. Semi-supervised classification with graph convolutional networks. In *International Conference on Learning Representations*, 2017.

[3] Vinod Nair and Geoffrey E Hinton. Rectified linear units improve restricted boltzmann machines. In *Proceedings of the 27th international conference on machine learning (ICML-10)*, pages 807–814, 2010.

[4] Nitish Srivastava, Geoffrey Hinton, Alex Krizhevsky, Ilya Sutskever, and Ruslan Salakhutdinov. Dropout: A simple way to prevent neural networks from overfitting. *Journal of Machine Learning Research*, 15(56):1929–1958, 2014.

[5] Emanuele Rossi, Henry Kenlay, Maria I Gorinova, Benjamin Paul Chamberlain, Xiaowen Dong, and Michael M Bronstein. On the unreasonable effectiveness of feature propagation in learning on graphs with missing node features. In *Learning on Graphs Conference*, pages 11–1. PMLR, 2022.

[6] Tianqi Chen and Carlos Guestrin. Xgboost: A scalable tree boosting system. In *Proceedings of the 22nd ACM SIGKDD International Conference on Knowledge Discovery and Data Mining*, KDD '16, page 785–794, New York, NY, USA, 2016. Association for Computing Machinery.

[7] F. Alexander Wolf, Philipp Angerer, and Fabian J. Theis. Scanpy: large-scale single-cell gene expression data analysis. *Genome Biology*, 19(1):15, 2018.

[8] Kevin Lebrigand, Joseph Bergenstråhle, Kim Thrane, Annelie Mollbrink, Konstantinos Meletis, Pascal Barbry, Rainer Waldmann, and Joakim Lundeberg. The spatial landscape of gene expression isoforms in tissue sections. *Nucleic Acids Research*, 51(8):e47–e47, 03 2023.

[9] Ao Chen, Sha Liao, Mengnan Cheng, Kailong Ma, Liang Wu, Yiwei Lai, Xiaojie Qiu, Jin Yang, Jiangshan Xu, Shijie Hao, Xin Wang, Huifang Lu, Xi Chen, Xing Liu, Xin Huang, Zhao Li, Yan Hong, Yujia Jiang, Jian Peng, Shuai Liu, Mengzhe Shen, Chuanyu Liu, Quanshui Li, Yue Yuan, Xiaoyu Wei, Huiwen Zheng, Weimin Feng, Zhifeng Wang, Yang Liu, Zhaohui Wang, Yunzhi Yang, Haitao Xiang, Lei Han, Baoming Qin, Pengcheng Guo, Guangyao Lai, Pura Muñoz-Cánoves, Patrick H. Maxwell, Jean Paul Thiery, Qing-Feng Wu, Fuxiang Zhao, Bichao Chen, Mei Li, Xi Dai, Shuai Wang, Haoyan Kuang, Junhou Hui, Liqun Wang,

Ji-Feng Fei, Ou Wang, Xiaofeng Wei, Haorong Lu, Bo Wang, Shiping Liu, Ying Gu, Ming Ni, Wenwei Zhang, Feng Mu, Ye Yin, Huanming Yang, Michael Lisby, Richard J. Cornall, Jan Mulder, Mathias Uhlén, Miguel A. Esteban, Yuxiang Li, Longqi Liu, Xun Xu, and Jian Wang. Spatiotemporal transcriptomic atlas of mouse organogenesis using dna nanoball-patterned arrays. *Cell*, 185(10):1777–1792.e21, 2022.

[10] Kangning Dong and Shihua Zhang. Deciphering spatial domains from spatially resolved transcriptomics with an adaptive graph attention auto-encoder. *Nature Communications*, 13(1):1739, 2022.

[11] Adam Paszke, Sam Gross, Francisco Massa, Adam Lerer, James Bradbury, Gregory Chanan, Trevor Killeen, Zeming Lin, Natalia Gimelshein, Luca Antiga, Alban Desmaison, Andreas Köpf, Edward Yang, Zach DeVito, Martin Raison, Alykhan Tejani, Sasank Chilamkurthy, Benoit Steiner, Lu Fang, Junjie Bai, and Soumith Chintala. *PyTorch: an imperative style, high-performance deep learning library.* Curran Associates Inc., Red Hook, NY, USA, 2019.

[12] V. A. Traag, L. Waltman, and N. J. van Eck. From louvain to leiden: guaranteeing well-connected communities. *Scientific Reports*, 9(1):5233, 2019.

[13] Zixuan Cang, Yanxiang Zhao, Axel A. Almet, Adam Stabell, Raul Ramos, Maksim V. Plikus, Scott X. Atwood, and Qing Nie. Screening cell–cell communication in spatial transcriptomics via collective optimal transport. *Nature Methods*, 20(2):218–228, 2023.

[14] Rohit Arora, Christian Cao, Mehul Kumar, Sarthak Sinha, Ayan Chanda, Reid McNeil, Divya Samuel, Rahul K Arora, T Wayne Matthews, Shamir Chandarana, et al. Spatial transcriptomics reveals distinct and conserved tumor core and edge architectures that predict survival and targeted therapy response. *Nature Communications*, 14(1):5029, 2023.

[15] Kristen R Maynard, Leonardo Collado-Torres, Lukas M Weber, Cedric Uytingco, Brianna K Barry, Stephen R Williams, Joseph L Catallini, Matthew N Tran, Zachary Besich, Madhavi Tippani, et al. Transcriptome-scale spatial gene expression in the human dorsolateral prefrontal cortex. *Nature neuroscience*, 24(3):425–436, 2021.

[16] Xinhao Liu, Ron Zeira, and Benjamin J Raphael. Paste2: partial alignment of multi-slice spatially resolved transcriptomics data. *bioRxiv*, 2023.

[17] Amanda Janesick, Robert Shelansky, Andrew D. Gottscho, Florian Wagner, Stephen R. Williams, Morgane Rouault, Ghezal Beliakoff, Carolyn A. Morrison, Michelli F. Oliveira, Jordan T. Sicherman, Andrew Kohlway, Jawad Abousoud, Tingsheng Yu Drennon, Seayar H. Mohabbat, Sarah E. B. Taylor, and 10x Development Teams. High resolution mapping of

the tumor microenvironment using integrated single-cell, spatial and in situ analysis. *Nature Communications*, 14(1):8353, 2023.

[18] Peter J. Rousseeuw. Silhouettes: A graphical aid to the interpretation and validation of cluster analysis. *Journal of Computational and Applied Mathematics*, 20:53–65, 1987.

[19] T. Caliński and J Harabasz. A dendrite method for cluster analysis. *Communications in Statistics*, 3(1):1–27, 1974.

[20] Ilya Korsunsky, Nghia Millard, Jean Fan, Kamil Slowikowski, Fan Zhang, Kevin Wei, Yuriy Baglaenko, Michael Brenner, Po-ru Loh, and Soumya Raychaudhuri. Fast, sensitive and accurate integration of single-cell data with harmony. *Nature methods*, 16(12):1289–1296, 2019.

[21] Yahui Long, Kok Siong Ang, Mengwei Li, Kian Long Kelvin Chong, Raman Sethi, Chengwei Zhong, Hang Xu, Zhiwei Ong, Karishma Sachaphibulkij, Ao Chen, Li Zeng, Huazhu Fu, Min Wu, Lina Hsiu Kim Lim, Longqi Liu, and Jinmiao Chen. Spatially informed clustering, integration, and deconvolution of spatial transcriptomics with graphst. *Nature Communications*, 14(1):1155, 2023.

[22] Edward Zhao, Matthew R. Stone, Xing Ren, Jamie Guenthoer, Kimberly S. Smythe, Thomas Pulliam, Stephen R. Williams, Cedric R. Uytingco, Sarah E. B. Taylor, Paul Nghiem, Jason H. Bielas, and Raphael Gottardo. Spatial transcriptomics at subspot resolution with bayesspace. *Nature Biotechnology*, 39(11):1375–1384, 2021.

[23] Chang Xu, Xiyun Jin, Songren Wei, Pingping Wang, Meng Luo, Zhaochun Xu, Wenyi Yang, Yideng Cai, Lixing Xiao, Xiaoyu Lin, Hongxin Liu, Rui Cheng, Fenglan Pang, Rui Chen, Xi Su, Ying Hu, Guohua Wang, and Qinghua Jiang. DeepST: identifying spatial domains in spatial transcriptomics by deep learning. *Nucleic Acids Research*, 50(22):e131–e131, 10 2022.

[24] Jian Hu, Xiangjie Li, Kyle Coleman, Amelia Schroeder, Nan Ma, David J. Irwin, Edward B. Lee, Russell T. Shinohara, and Mingyao Li. Spagcn: Integrating gene expression, spatial location and histology to identify spatial domains and spatially variable genes by graph convolutional network. *Nature Methods*, 18(11):1342–1351, 2021.

[25] Lauren L Hsu and Aedín C Culhane. Correspondence analysis for dimension reduction, batch integration, and visualization of single-cell rna-seq data. *Scientific Reports*, 13(1):1197, 2023.

[26] Romain Lopez, Jeffrey Regier, Michael B. Cole, Michael I. Jordan, and Nir Yosef. Deep generative modeling for single-cell transcriptomics. *Nature Methods*, 15(12):1053–1058, 2018.

[27] Vipul Singhal, Nigel Chou, Joseph Lee, Yifei Yue, Jinyue Liu, Wan Kee Chock, Li Lin, Yun-Ching Chang, Erica Mei Ling Teo, Jonathan Aow, et al. Banksy unifies cell typing and tissue domain segmentation for scalable spatial omics data analysis. *Nature genetics*, 56(3):431–441, 2024.

[28] Tian Tian, Jie Zhang, Xiang Lin, Zhi Wei, and Hakon Hakonarson. Dependency-aware deep generative models for multitasking analysis of spatial omics data. *Nature Methods*, 21(8):1501–1513, 2024.
